# Remote and Selective Control of Astrocytes by Magnetomechanical Stimulation

**DOI:** 10.1002/advs.202104194

**Published:** 2021-12-19

**Authors:** Yichao Yu, Christopher Payne, Nephtali Marina, Alla Korsak, Paul Southern, Ana García‐Prieto, Isabel N. Christie, Rebecca R. Baker, Elizabeth M. C. Fisher, Jack A. Wells, Tammy L. Kalber, Quentin A. Pankhurst, Alexander V. Gourine, Mark F. Lythgoe

**Affiliations:** ^1^ Centre for Advanced Biomedical Imaging Division of Medicine University College London 72 Huntley Street London WC1E 6DD UK; ^2^ Centre for Cardiovascular and Metabolic Neuroscience Research Department of Neuroscience, Physiology and Pharmacology University College London Gower Street London WC1E 6BT UK; ^3^ Healthcare Biomagnetics Laboratory University College London 21 Albemarle Street London W1S 4BS UK; ^4^ Departamento Física Aplicada I Universidad del País Vasco Bilbao 48013 Spain; ^5^ Department of Neuromuscular Diseases Queen Square Institute of Neurology University College London Queen Square London WC1N 3BG UK

**Keywords:** astrocytes, adenosine triphosphate, calcium, iron oxide particles, magnetic actuation, magnetomechanical stimulation

## Abstract

Astrocytes play crucial and diverse roles in brain health and disease. The ability to selectively control astrocytes provides a valuable tool for understanding their function and has the therapeutic potential to correct dysfunction. Existing technologies such as optogenetics and chemogenetics require the introduction of foreign proteins, which adds a layer of complication and hinders their clinical translation. A novel technique, magnetomechanical stimulation (MMS), that enables remote and selective control of astrocytes without genetic modification is described here. MMS exploits the mechanosensitivity of astrocytes and triggers mechanogated Ca^2+^ and adenosine triphosphate (ATP) signaling by applying a magnetic field to antibody‐functionalized magnetic particles that are targeted to astrocytes. Using purpose‐built magnetic devices, the mechanosensory threshold of astrocytes is determined, a sub‐micrometer particle for effective MMS is identified, the in vivo fate of the particles is established, and cardiovascular responses are induced in rats after particles are delivered to specific brainstem astrocytes. By eliminating the need for device implantation and genetic modification, MMS is a method for controlling astroglial activity with an improved prospect for clinical application than existing technologies.

## Graphical abstract

Magnetomechanical stimulation (MMS) is a novel technology that enables remote and selective control of brain cell activity without any need for genetic modification or device implantation. Antibody‐functionalized magnetic particles are targeted to the mechanosensitive astrocytes and a magnetic field is applied to activate Ca^2+^ and adenosine triphosphate (ATP) signaling that regulate a wide range of physiological, cognitive, and behavioral processes.

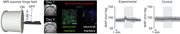

## 1 Introduction

Astrocytes are a major type of glial cells in the central nervous system (CNS). They constitute an integral part of neural circuitry,^[^ ^1^ ^]^ regulate a wide range of homeostatic processes,^[^ ^2^ ^]^ and play key roles in the brain's defense against disease and injury.^[^ ^3^ ^]^ Due to their extensive involvement in CNS function, astrocytes are implicated in many neurological disorders including neurodegenerative diseases,^[^ ^4^ ^]^ epilepsy^[^ ^4^ ^]^ and stroke.^[^ ^5^ ^]^ In recent years, it has become possible to modulate astroglial activity with spatial, temporal and cell type specificity thanks to the advent of cell control methods such as optogenetics and chemogenetics.^[^ ^6^ ^]^ These technological advances have not only provided better tools to study these important cells in health and disease,^[^ ^4^, ^6^ ^]^ but also opened new avenues for therapy development.^[^ ^7^, ^8^, ^9^ ^]^ For example, optogenetics has been used to introduce a light‐gated ion channel such as channelrhodopsin‐2 (ChR2) into astrocytes and enable photostimulation of intracellular Ca^2+^ signaling and adenosine triphosphate (ATP) release, thereby helping researchers to elucidate how astrocytes regulate respiration^[^ ^10^ ^]^ and pain sensitivity.^[^ ^11^ ^]^ Chemogenetics involves using a designer drug to specifically activate an exogenous designer G protein‐coupled receptor and it has been employed to demonstrate astroglial regulation of feeding behavior^[^ ^12^ ^]^ and fear conditioning^[^ ^13^ ^]^ through ATP/adenosine signaling. Optogenetic and chemogenetic manipulations of astrocytes have also been explored as therapies for conditions such as epilepsy,^[^ ^7^ ^]^ drug seeking behavior,^[^ ^8^ ^]^ and neurodegenerative diseases.^[^ ^9^ ^]^

Though powerful, optogenetics and chemogenetics are not without limitations. Optogenetics usually requires optic fibers to be implanted into the brain, making it highly invasive. With chemogenetics, the designer drug is administered systemically and slow‐acting, therefore this method is unsuitable for engaging fast and precise dynamics. An alternative approach is magnetogenetics, which utilizes virally transduced temperature‐ and/or mechanogated Transient Receptor Potential (TRP) channels such as TRPV1 and TRPV4 to enable control of cell activity with a magnetic field.^[^ ^14^, ^15^, ^16^, ^17^, ^18^, ^19^ ^]^ The action is mediated by magnetic nanoparticles (MNPs) that are attached to the TRP channels, and two strategies have been employed to trigger channel opening. One is to use a temperature‐sensitive TRP channel, which can be activated by heating the attached MNPs with a radio‐frequency alternating magnetic field (AMF).^[^ ^14^, ^15^, ^16^, ^17^, ^18^ ^]^ The other is to use a mechanogated TRP channel, which can be opened by exerting force on the attached MNPs with an inhomogeneous magnetic field.^[^ ^16^, ^17^, ^19^ ^]^ Magnetogenetics allows remote control because magnetic fields suffer very little attenuation by biological tissue, therefore can be applied to deep structures from the outside. However, there are concerns that magnetogenetic control of cell function is difficult to achieve^[^ ^20^ ^]^ and the underlying mechanisms are unclear.^[^ ^21^ ^]^ Furthermore, optogenetics, chemogenetics and magnetogenetics all require the expression of an exogenous protein in the cells of interest, an intervention that has unclear safety consequences, especially in the long term, and represents a major hurdle to the translation of these technologies into the clinic.^[^ ^22^, ^23^ ^]^

Here, we introduce magnetomechanical stimulation (MMS) as a magnetism‐based, selective cell control technology that does not involve genetic modification and therefore confers the advantage of remote control plus the additional benefit of simplicity in terms of its usability and the underlying mechanism. Sensitivity to mechanical stimuli is an endogenous feature of astrocytes. For instance, touching astrocytes in culture with a micropipette causes elevations in intracellular Ca^2+^ concentration ([Ca^2+^]_i_)^[^ ^24^, ^25^ ^]^ and ATP release.^[^ ^26^, ^27^ ^]^ Exploiting such mechanosensitivity, we demonstrate the feasibility of remotely triggering astroglial Ca^2+^ and ATP signaling by MMS, which involves targeting antibody‐functionalized magnetic iron oxide particles to the astrocytes and applying a magnetic field to exert forces on the particles and mechanically stimulate the cells. Besides being a useful research tool for dissecting the functional roles of astrocytes in neural circuits, the obviation of the need for genetic modification would also make MMS a more attractive candidate for clinical translation than the genetic methods.

To develop MMS, we first determined the minimum mechanical stimuli required to trigger Ca^2+^ and ATP signaling in astrocytes using a bespoke device that is capable of applying precise forces. Then we assessed a series of magnetic particles and selected a sub‐micrometer one that enabled effective and selective MMS, after investigating various factors including the size and magnetic properties of the particles, the degree of particle aggregation on cells, and the type of ligand conjugated to the particles. Next we designed and tested a magnetic device that could produce the required mechanical stimuli across the brain of a small rodent. Finally, using either the purpose‐built magnetic device or the fringe magnetic field of an MRI scanner to actuate particles conjugated with an astrocyte‐specific antibody, we confirmed that MMS of a specific group of brainstem astrocytes evoked expected cardiovascular responses in rats, thereby providing proof of concept.

## 2 Results

### 2.1 Mechanosensory Threshold of Astrocytes in Culture

In order to guide magnetic particle selection and magnetic device design for in vivo applications, we first determined the minimum mechanical stimulus required to elicit Ca^2+^ and ATP signaling responses from astrocytes in culture.

**Figure 1:**
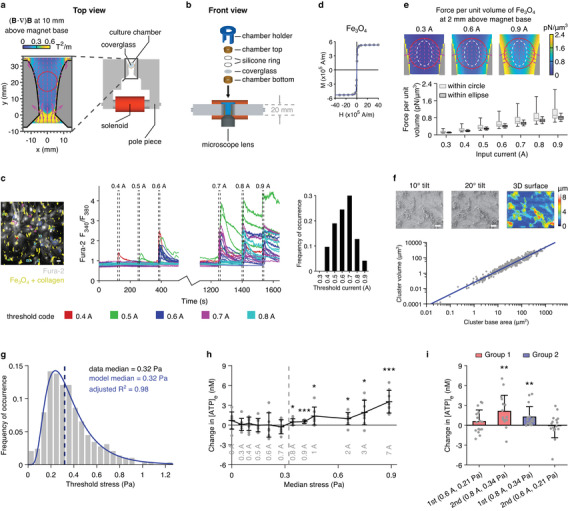
Threshold of astroglial sensitivity to mechanical stimuli. a) Design of the yoke magnet. Coverglass diameter = 12 mm. Blow‐up: the shape of the pole pieces is such that (***B*** · ∇) ***B***, which predicts the force exerted on magnetic particles of the same type and size, is highly uniform within a large region of the inter‐pole space (red circle). b) Front view of the experimental setup. c) An example field of view (FOV) showing astrocytes with collagen‐coated Fe_3_O_4_ particles (left) and the Fura‐2 fluorescence ratiometric traces derived from it (middle). The traces and the regions of interest (ROIs) are colour‐coded according to the minimum input currents required to trigger Ca^2+^ signals in the corresponding cells. The threshold currents obtained from 290 cells are summarized in the right panel. d) Magnetization curve of Fe_3_O_4_ particles. e) Simulation of the force exerted on a unit volume of Fe_3_O_4_ particles at different input currents. Top: example maps; red circle = cell culture location; white dashed ellipse = region of high uniformity where Ca^2+^ imaging was restricted to. Bottom: summary statistics; *n* = 441 points for each “within circle” condition; *n* = 184 points for each “within ellipse” condition; bar, median; box, quartiles; whiskers, range. f) SEM images of astrocyte cultures taken at different tilt angles of the stage (top left and top middle) were used for stereoscopic 3D surface reconstruction (top right) and calculation of the volume and base area of Fe_3_O_4_ clusters (*n* = 401, bottom), which have a well‐defined relationship. g) The threshold stresses for triggering Ca^2+^ signals in individual astrocytes (*n* = 290) were calculated from Panel c, e and f and they exhibit a lognormal distribution. h) Changes in extracellular ATP concentration ([ATP]_e_) following MMS. Horizontal coordinate indicates the median of the stresses experienced by the whole culture (Figure S2i, Supporting Information). *n* = 8 measurements for each condition. i) Repeated testing of the same cultures. *n* = 16 cultures for each condition. In Panel (c,f): scale bar = 20 µm. In Panel (h,i): data shown as mean ± standard deviation (S.D.); *, *p* < 0.05, two‐tailed *t*‐test of mean [ATP]_e_ change equaling zero; **, *p* < 0.01, same test; ***, *p* < 0.001, same test.

We designed a “yoke” electromagnet that is compatible with a conventional optical microscope and capable of applying a precise mechanical stimulus to multiple cells through magnetic particles (**Figure** **1**a,b). With this actuation device and collagen‐coated magnetite (Fe_3_O_4_) particles, we determined that the minimum mechanical stress required to induce a [Ca^2+^]_i_ response in astrocytes was 0.32 Pa (Figure 1g). Three sets of data were obtained to enable this estimation. First, we increased the force applied to particle‐adorned astrocytes by stepping up the current supplied to the magnet and determined that the minimum input current required to trigger a Ca^2+^ signal in individual cells was between 0.4 and 0.9 A (Figure 1c), corresponding to a median magnetic flux density between 0.033 and 0.071 T. Second, we calculated the force exerted on a unit volume of Fe_3_O_4_ particles for each input current that had been used (e.g., 0.31–0.52 pN µm^−3^ within the central region of the culture for 0.6 A, Figure 1e), taking into account both the magnetic field generated by the device (Figure S2a, Supporting Information) and the magnetization curve of the particles (Figure 1d). Lastly, because the Fe_3_O_4_ clusters attached to the astrocytes were irregularly shaped and variable in size (Figure 1c, left panel), we estimated their volumes individually as follows. We performed scanning electron microscopy (SEM) and stereoscopic surface reconstruction on several cultures and established that there was a well‐defined power law relationship between the base areas and volumes of Fe_3_O_4_ clusters (Figure 1f). Using this relationship, we could indirectly estimate the volumes of the Fe_3_O_4_ clusters seen in bright‐field micrographs (Figure 1c, left panel), since their base areas were known. Combining the minimum input current required to trigger a response in a given cell, the calculated force applied to a unit volume of Fe_3_O_4_ particles at that input current, and the volume of the Fe_3_O_4_ cluster attached to that cell, we could calculate the threshold force for inducing a Ca^2+^ signal in that cell. We determined the threshold forces for 290 astrocytes, which have a median of 40.06 pN (Figure S2f, Supporting Information). From these threshold forces we derived threshold stresses, a more relevant determinant of cell membrane deformation. The threshold stress values have a median of 0.32 Pa and exhibit a lognormal distribution (Figure 1g).

As an additional measure of the astrocytes’ response to mechanical stimulation, and because the release of ATP is the central event in the astroglial mechanosensory signaling cascade,^[^ ^28^ ^]^ we also quantified changes in extracellular ATP concentration ([ATP]_e_) following MMS. A significant increase in [ATP]_e_ was only recorded when the magnet was driven by a current equal to or greater than 0.8 A (Figure 1h), generating a median magnetic flux density equal to or greater than 0.064 T. Repeatedly testing the same cultures also showed that, irrespective of the order of the stimulations, an input current of 0.8 A was required to reliably induce ATP release while a 0.6 A current, corresponding to a median magnetic flux density of 0.050 T, was ineffective (Figure 1i). These results corroborate those obtained in the Ca^2+^ imaging experiments, because the median of the stresses experienced by the astrocyte population was estimated to surpass the 0.32 Pa threshold when the input current was 0.8 A (median stress equals 0.28 Pa at 0.7 A and 0.34 Pa at 0.8 A, Figure S2i, Supporting Information).

### 2.2 Selective MMS of Astrocytes In Vitro

**Figure 2:**
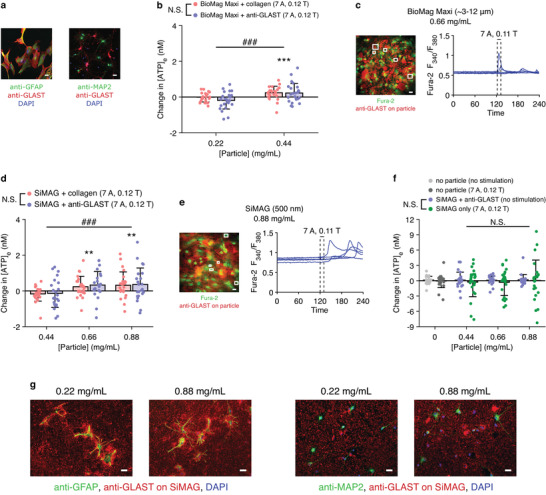
Particles with adequate mechanical actuation potential and binding specificity. a) The anti‐GLAST antibody labeled cells expressing the astroglial marker glial fibrillary acidic protein (GFAP), but not those expressing the neuronal marker microtubule‐associated protein 2 (MAP2). b) Magnetic actuation of BioMag Maxi particles on cultured astrocytes by the yoke magnet (7 A, 10 mm above base, median magnetic flux density = 0.12 T) induced ATP release when the concentration of the particle suspension applied to the cells ([particle]) was 0.44 mg mL^−1^, whereas no [ATP]_e_ change was observed when [particle] was 0.22 mg mL^−1^ ([particle] effect, ###, *p* < 0.001). The type of ligand on the particles (collagen versus anti‐GLAST) did not affect [ATP]_e_ changes (N.S., not significant, *p* = 0.278). See Table S2 in the Supporting Information. c) Magnetic actuation (7 A, 2 mm above base, median magnetic flux density = 0.11 T) of anti‐GLAST‐coupled BioMag Maxi particles ([particle] = 0.66 mg mL^−1^) on cultured astrocytes triggered Ca^2+^ signals. d,e) Same experiments with SiMAG particles. Similarly, [particle] (###, *p* < 0.001), but not the type of ligand on the particles (N.S., *p* = 0.641), had a significant effect on [ATP]_e_ changes (Table S3, Supporting Information). f) Control experiments. Neither [particle] (N.S., *p* = 0.689) nor the stimulation regime (N.S., *p* = 0.427) had a significant effect on [ATP]_e_ changes (Table S4, Supporting Information). g) Anti‐GLAST‐coupled SiMAG particles showed higher binding affinity for GFAP‐positive astrocytes in comparison to MAP2‐positive neurons. In Panel (c,e,g): scale bar = 20 µm. In Panel (b,d,f): data shown as mean ± S.D.; *n* = 24 measurements for each condition; **, *p* < 0.01, two‐tailed *t*‐test of mean [ATP]_e_ change equaling zero; ***, *p* < 0.001, same test.

We next explored the possibility of selectively stimulating astrocytes using iron oxide particles functionalized with the monoclonal anti‐GLAST antibody,^[^ ^29^ ^]^ which binds to an extracellular epitope of the astrocyte‐specific membrane protein glutamate‐aspartate transporter (GLAST) (**Figure** **2**a).

We evaluated four types of particles whose nominal sizes range from 100 nm to over 10 µm (Table S1, Supporting Information), obtaining images and the magnetization curve of each type (Figure S3a,b, Supporting Information). Subsequent calculations indicated that none of them, when sparsely attached to astrocytes and actuated by the yoke magnet, would generate a median stress greater than the 0.32 Pa threshold (Figure S3c, Supporting Information). Indeed, when BioMag Plus (1.5 µm) or BioMag Maxi (3–12 µm) particles were applied to astrocyte cultures at a low concentration (0.22 mg mL^−1^), no ATP release was observed following MMS with the yoke magnet (Figure S3d, Supporting Information; Figure 2b). As individual particles—even very large ones—were ineffective, we sought to enhance particle aggregation by incubating cells with more concentrated suspensions (Figure S3e, Supporting Information), so that a cluster of multiple particles would act as a single unit with an increased volume. After applying BioMag Maxi particles to astrocytes at higher concentrations, we observed clear MMS‐induced ATP release (0.44 mg mL^−1^, *p* = 0.00090, Figure 2b) and Ca^2+^ signals (0.66 mg mL^−1^, Figure 2c). The measured [ATP]_e_ changes were similar between experiments using collagen‐coated particles and those using anti‐GLAST‐coupled ones (*p* = 0.278, Figure 2b). This was likely because both types of particles bound to the high purity astrocyte cultures (Figure S1, Supporting Information) in similar numbers.

Since the large BioMag Maxi particles would be impractical for in vivo applications, we next investigated the feasibility of using the smaller SiMAG particles (500 nm). MMS with the yoke magnet induced ATP release from astrocyte cultures that had been incubated with SiMAG particles at a concentration of either 0.88 mg mL^−1^ (*p* = 0.0062) or 0.66 mg mL^−1^ (*p* = 0.0054), but not 0.44 mg mL^−1^ (*p* = 0.091) (Figure 2d). Moreover, particle concentration had a strong positive effect on the amount of ATP released (*p* = 0.00089), while the type of ligand (collagen versus anti‐GLAST) on the particles did not affect the results (*p* = 0.64) (Figure 2d). We also recorded MMS‐induced Ca^2+^ signals in astrocytes after applying anti‐GLAST‐coupled SiMAG particles at 0.88 mg mL^−1^ (Figure 2e). In contrast, no astroglial ATP release was observed in four control experiments (Figure 2f): i) no particles were used and no magnetic field was applied; ii) no particles were used but a magnetic field was applied, suggesting that the astrocytes were not affected by the magnetic field itself; iii) anti‐GLAST‐coupled SiMAG particles were attached to the cells, but no magnetic field was applied; and iv) unmodified SiMAG particles were attached to the cells and a magnetic field was applied. Importantly, increasing the particle concentration did not affect the [ATP]_e_ changes recorded in these control experiments (*p* = 0.69, Figure 2f), meaning that the presence of more particles on cells, in itself, did not contribute to the increased ATP release seen in previous experiments. Finally, immunocytochemistry of mixed neural cell cultures showed that anti‐GLAST‐coupled SiMAG particles were attached predominantly to astrocytes (Figure 2g). Taken together, these results demonstrate that Ca^2+^ and ATP signaling responses could be induced in cultured astrocytes with a high degree of specificity by magnetomechanical actuation of SiMAG particles functionalized with the anti‐GLAST antibody.

### 2.3 Magnetic Devices for In Vivo Applications

After particle assessment and selection, we developed two magnetic devices that could apply a magnetic field across the rodent brain in vivo and produce greater mechanical stimuli than the yoke magnet.

**Figure 3:**
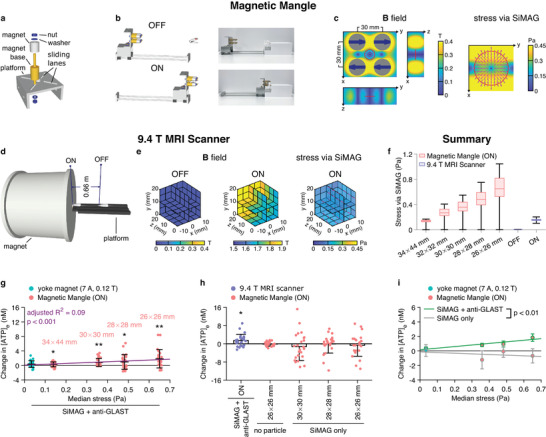
Design and testing of magnetic devices. a) Design of the Magnetic Mangle. The orientations and positions of the magnets are adjustable. b) Schematic of the in vivo experimental setup (left) and photographs of the Magnetic Mangle and the animal bed (right). c) Maps showing simulated magnetic field produced by the Magnetic Mangle and estimated stress via SiMAG particles. Gray closed circles represent magnets. Indigo arrows indicate magnetization directions. Red open circles and lines indicate the cell culture location for in vitro testing. d) Fringe field of an MRI scanner used for MMS. e) Magnetic field was measured at both the “OFF” and “ON” positions (left and middle), and stress via SiMAG particles was estimated (right). f) Summary statistics of the estimated stresses via SiMAG particles within the cell culture location for the Magnetic Mangle (*n* = 441 points for each condition) and within the whole measurement cube for the MRI scanner (*n* = 125 points for each condition). Bar, median. Box, quartiles. Whiskers, range. g–i) [ATP]_e_ changes after MMS of astrocytes in culture with different devices and SiMAG preparations. In Panel (g,h): data shown as mean ± S.D.; purple line in Panel (g), linear regression (Table S7, Supporting Information). In Panel (i): data shown as mean ± standard error; using anti‐GLAST‐coupled SiMAG particles resulted in greater MMS‐induced ATP release than using unmodified SiMAG particles (Table S8, Supporting Information). In Panel (g‐i): *n* = 24 measurements for each condition; *, *p* < 0.05, two‐tailed *t*‐test of mean [ATP]_e_ change equaling zero; **, *p* < 0.01, same test.

The Magnetic Mangle consisted of four diametrically magnetized ring magnets arranged in a rectangular grid on a platform (**Figure** **3**a–c). By mounting the platform on a track, the magnets could be moved between the “on” position where they would surround a rodent's head and the “off” position about 0.3 m away from the animal (Figure 3b). The magnetic field in the inter‐magnet space could be manipulated by altering the orientations of the magnets’ poles or adjusting the distances between the magnets. We first compared 4 different ways of arranging the magnets’ orientations (Figure S4a–d, Supporting Information) and selected one that generated the highest stress (Configuration 4 in Figure S4a, Supporting Information) for use in subsequent experiments. We then established with modeling that bringing the magnets closer to each other would produce higher stresses (Figure 3f) and verified with in vitro testing that actuation of anti‐GLAST‐coupled SiMAG particles attached to astrocytes with a smaller magnet grid resulted in more ATP release (Figure 3g). Taking results from both the yoke magnet and the Magnetic Mangle into account, there was a strong positive correlation between the estimated stress output and the measured changes in [ATP]_e_ (*p* = 0.00058, Figure 3g). In the control experiments where no particles or unmodified SiMAG particles were used, no astroglial ATP release was recorded in response to MMS (Figure 3h) and there was a significant difference between results obtained using unmodified SiMAG particles and those measured using anti‐GLAST‐coupled SiMAG particles (*p* = 0.0053, Figure 3i).

We also evaluated the fringe field of a 9.4 T MRI scanner as a device for MMS. The edge of the horizontal magnet bore and a location 0.66 m away from the edge were picked as “on” and “off” positions respectively (Figure 3d). Magnetic field measurements and subsequent calculations (Figure 3e) indicated that the scanner fringe field would exert lower stresses than the Magnetic Mangle when the magnet grid of the latter was 32 mm by 32 mm or smaller (Figure 3f). This is because, in comparison to the Magnetic Mangle, the increase in particle magnetization due to the higher field strength of the MRI scanner fringe field (Figure S4e,f, Supporting Information) could not overcome the decrease in field gradient (Figure S4g,h, Supporting Information). ATP release was observed after astrocyte cultures with anti‐GLAST‐coupled SiMAG particles were exposed to the scanner fringe field (*p* = 0.015, Figure 3h).

### 2.4 Fate of the Particles In Vivo

Astrocytes in the ventrolateral medulla (VLM) are able to modulate the activity of the local tyrosine hydroxylase (TH)‐expressing sympathoexcitatory C1 neurons via ATP‐mediated signaling, and produce increases in central sympathetic drive, arterial blood pressure (ABP) and heart rate.^[^ ^30^, ^31^ ^]^ To validate the method of MMS in vivo, we aimed to stimulate the astrocytes in the VLM of rats, while monitoring changes in ABP as an indicator of astroglial signaling in that brain region. To prepare for these experiments, we investigated the fate of anti‐GLAST‐coupled SiMAG particles after they were injected into the rat brainstem.

**Figure 4:**
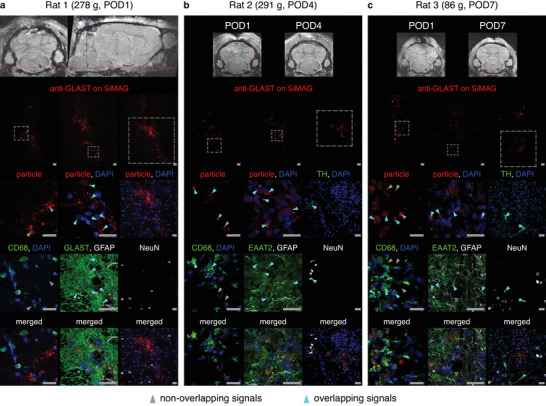
Fate of anti‐GLAST‐coupled SiMAG particles injected into the rat brainstem. Each rat received a unilateral 1 µL injection of a 0.25 mg mL^−1^ suspension of anti‐GLAST‐coupled SiMAG particles. MRI was performed and the acquired images were affinely registered to each other. The cross‐hairs in MR images mark the same anatomical location in each brain. Animals were sacrificed at different time points and brainstem sections were stained for microglial (CD68), astroglial (GFAP, GLAST, and EAAT2), and neuronal (NeuN and TH) markers. Three consecutive sections are shown for each rat. POD, postoperative day. Scale bar = 20 µm. Number of animals used = 3.

We used MRI to determine the location of the injection site, followed by immunohistochemistry to confirm that the particles were in close proximity to TH‐positive neurons (**Figure** **4** **;** Figure S5, Supporting Information). One day after injecting 1 µL of a suspension with a concentration of either 1 mg mL^−1^ or 0.5 mg mL^−1^, we observed conspicuous aggregation of the particles (Figure S5a,b, Supporting Information). The distribution was more diffuse when a 0.25 mg mL^−1^ suspension was microinjected (Figure 4a). The particles remained visible in MR images at postoperative day (POD) 4 and POD7, but fluorescence signals from the particles became progressively weaker at POD4 and POD7 (Figure 4b,c; Figure S5c, Supporting Information), suggesting that some particles were cleared over this time. Anti‐GLAST‐coupled SiMAG particles were found to colocalize with or be in the immediate vicinity of astrocytes, which were labeled with an antibody against glial fibrillary acidic protein (GFAP) and an antibody against either excitatory amino acid transporter 2 (EAAT2) or GLAST, whereas the nuclei of neurons, labeled with an antibody against neuronal nuclei (NeuN), were mostly at a distance from the particles (Figure 4; Figure S5, Supporting Information). The invading microglia, labeled with an antibody against cluster of differentiation 68 (CD68), showed virtually no overlap with signals from the particles at POD1, but at POD4 and POD7, some particles could be seen to colocalize with these cells (Figure 4; Figure S5, Supporting Information).

These findings demonstrate that anti‐GLAST‐coupled SiMAG particles injected into the rat brainstem bind to astrocytes. The distribution and retention of the particles appear to be sensitive to the concentration of the particles in the injection suspension and the passage of time. For the subsequent functional studies, we used particle suspensions with a concentration of either 0.25 or 0.5 mg mL^−1^ and performed the experiments within 4 days after the microinjections.

### 2.5 Magnetomechanical Stimulation of Astrocytes In Vivo

**Figure 5:**
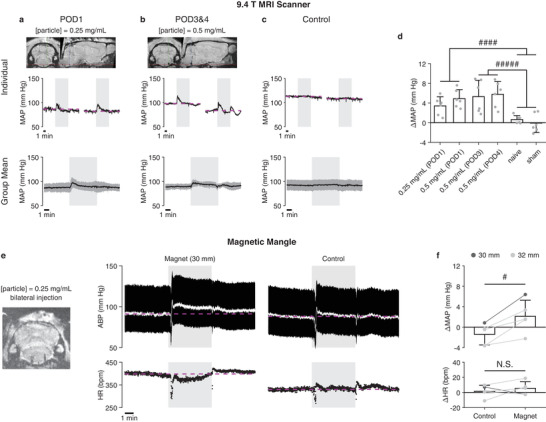
Magnetomechanical stimulation of astrocytes in vivo. a–c) Cardiovascular responses in anesthetized rats induced by MMS. The fringe magnetic field of the MRI scanner was applied by moving the animal's head to the edge of the scanner bore. Animals in experimental groups (a,b) were given unilateral microinjections of anti‐GLAST‐coupled SiMAG particles (1 µL, 0.25 or 0.50 mg mL^−1^) into the VLM and stimulation experiments were performed 1, 3, or 4 days after the injection. Control groups (c) consisted of naive animals and sham‐operated ones that received unilateral microinjections of an anti‐GLAST antibody solution (1 µL, 5 µg mL^−1^). Top and middle rows: data from an example animal of the group. Bottom row: average of the mean arterial pressure (MAP) traces; error bar = S.D.; *n* = 12 traces for each group. d) Summary statistics of the MAP changes (*Δ*MAP, the difference in MAP between the pre‐stimulation period and the period of magnetic field application, excluding the initial 30 s of cradle movement). *n* = 6 measurements for each condition (2 measurements per animal). Number of animals used = 18. ####, *p* < 0.0001, two‐sample two‐tailed *t*‐test. #####, *p* < 0.00001, same test. e) Cardiovascular responses induced by the Magnetic Mangle in a rat that received bilateral microinjections of anti‐GLAST‐coupled SiMAG particles into the VLM. For the control experiments, magnets were replaced with identically shaped plastic dummies so that the device would apply no magnetic field. White trace, MAP. ABP, arterial blood pressure. HR, heart rate. bpm, beats per minute. f) Summary statistics of changes in MAP and HR. *n* = 5 measurements for each condition (1 measurement per animal). Number of animals used = 5. #, *p* < 0.05, two‐tailed paired *t*‐test. In Panel (a–c,e): magenta dashed line, pre‐stimulation mean level. In Panel (d,f): data shown as mean ± S.D.

Initially, the MRI scanner was used as a magnetic actuation device. One, three or four days after a single 1 µL injection of anti‐GLAST‐coupled SiMAG particles (0.5 or 0.25 mg mL^−1^) in the VLM, we were able to repeatedly evoke a sharp rise in the ABP by moving the animal's head to the “on” position in the MRI scanner fringe field (**Figure** **5**a,b). In control groups (naive and sham‐operated animals), such ABP elevations were not observed following exposure of the head to the “on” position (Figure 5c). When compared, ABP changes were significantly greater in the experimental groups than in the control groups (*p* = 0.000013 for POD1 groups versus control groups, *p* = 0.0000096 for POD3 and POD4 groups versus control groups, Figure 5d).

Our benchtop device, the Magnetic Mangle, was used next and animals were given bilateral microinjections of anti‐GLAST‐coupled SiMAG particles. Application of the magnetic field triggered a sustained ABP elevation that lasted for more than 10 min (Figure 5e). For the control experiment, the ring magnets were replaced with identically shaped plastic dummies so that the device would apply no magnetic field. Moving the dummies to the “on” position did not cause the ABP to deviate from the baseline except for a short‐lasting initial peak (Figure 5e), which was likely due to the brief perturbation as the dummies came into contact with the animal's head. Overall, applications of a magnetic field led to significantly more positive ABP changes than applications of dummies (*p* = 0.013, Figure 5f), showing that the cardiovascular response was driven by MMS of the VLM astrocytes.

Taken together, these results demonstrated that in vivo control of astroglial signaling in a discrete area of the brain can be achieved with remote MMS.

## 3 Discussion

In this study we exploited the mechanosensitivity of astrocytes to develop a novel technology for remotely activating key astroglial signaling events. We established the mechanosensory threshold of astrocytes, assessed different types of iron oxide particles and magnetic devices, characterized the in vivo fate of the selected magnetic particles, and demonstrated the efficacy of MMS for remote control of astrocytes in the living brain.

In order to gauge the magnitude of the mechanical stimuli that need to be produced, we first investigated the mechanosensory threshold of astrocytes and found that the minimum stress required to trigger astroglial Ca^2+^ and ATP signaling was 0.32 Pa. Several studies in which cell cultures were subjected to shear stresses generated by fluid flow have reported similar thresholds for triggering Ca^2+^ signals (0.20 Pa,^[^ ^32^ ^]^ 0.23 Pa^[^ ^33^ ^]^ and 1.53 Pa^[^ ^34^ ^]^). The higher threshold of 1.53 Pa reported on astrocytes^[^ ^34^ ^]^ could be due to the fact that fluid flow applies shear stress to the entire apical surface of the cultured cell, whereas the stress generated via magnetic particles is much more localized. Moreover, mechanically stimulated Ca^2+^ signals in astrocytes largely depend on the autocrine actions of the released ATP.^[^ ^28^, ^35^ ^]^ Therefore when fluid flow is used as the stimulus, some of the ATP released may have been rapidly washed away, resulting in smaller responses and an overestimation of the threshold.

Next, we showed that the sub‐micrometer SiMAG particles, when functionalized with the anti‐GLAST antibody, bound selectively to astrocytes and enabled effective MMS. Noticeably, anti‐GLAST‐coupled particles performed as well as those coated with collagen, suggesting that the molecular identity of the particles’ target is unimportant and MMS is a generalizable method. Recently it has been shown that attaching magnetic particles to neurons in culture enabled them to be activated by a magnetic field.^[^ ^36^, ^37^ ^]^ Therefore, with a suitable targeting strategy, it is possible to achieve selective in vivo manipulation of neurons with MMS.

To demonstrate the feasibility of magnetomechanical stimulation of astrocytes in vivo, we utilized either the fringe field of an MRI scanner or a bespoke device.^[^ ^38^ ^]^ The latter, named the Magnetic Mangle, is a simple device that is inexpensive to make and capable of producing the required forces in small animals. With the MRI scanner fringe field, the limitations on the size of the subjects are mitigated and the scope of the study can be broadened to include behavioral experiments. For example, the fringe field of an MRI scanner has been used to navigate instruments through the pig vasculature,^[^ ^39^ ^]^ and modulate feeding behavior in free‐moving mice.^[^ ^17^ ^]^ Furthermore, the programmable gradients inside the MRI scanner bore have been used to steer magnetic therapeutic agents to improve delivery,^[^ ^40^, ^41^, ^42^ ^]^ and could be used in tandem with MMS.

The MMS technology lends itself well to clinical translation because of several distinct advantages. First, the obviation of genetic modification overcomes a major obstacle to developing optogenetics and chemogenetics as therapies.^[^ ^22^, ^23^ ^]^ Expressing an exogenous protein using a viral vector carries the general risks of insertional mutagenesis, oncogene activation and so on, as well as particular ones posed by the foreign protein itself. For instance, ChR2 activation permits proton influx, which in the case of astrocytes is highly undesirable because glial acidosis can lead to neuronal excitotoxicity.^[^ ^43^ ^]^ With MMS, such risks are non‐existent. Second, it may only require minimal development to leverage existing MRI scanners in hospitals as both an imager to non‐invasively evaluate particle delivery and the actuation device to perform MMS. Lastly, the safety of iron oxide nanoparticles (IONPs) has been extensively studied, both preclinically and clinically, for over 20 years.^[^ ^44^ ^]^ Studies have shown that IONPs have a high degree of biocompatibility in the brain^[^ ^45^ ^]^ and are well tolerated by astrocytes and neurons.^[^ ^46^, ^47^, ^48^ ^]^ In particular, astrocytes exhibited no substantial change in viability and glucose and glutathione metabolism up to a week after exposure to IONPs.^[^ ^49^ ^]^ In the live brain, directly injected IONPs were still present after 3 months and histological assessment revealed no pathological brain cell or myelin changes.^[^ ^50^ ^]^ Moreover, clinical magnetic hyperthermia trials, which involved intracranial injection of large amounts of IONPs, reported no serious side effects due to the particles.^[^ ^51^, ^52^, ^53^ ^]^ The clearance of the magnetic particles in the brain may involve microglia, as we noted some internalization of the particles by CD68‐positive cells. There is also evidence that the cervical lymph nodes are a possible clearance pathway for IONPs delivered to the brain parenchyma.^[^ ^54^ ^]^ Although previous studies indicate a robust safety profile, it must be noted that the uptake, stability and clearance, hence toxicity of IONPs are strongly affected by their physical and chemical properties;^[^ ^44^, ^45^ ^]^ therefore, the biocompatibility of the specific particles must be investigated before clinical application can be considered.

In comparison to existing methods of neuromodulation, MMS presents several challenges. One powerful feature of optogenetics is that it allows fast and precise control of neurons.^[^ ^55^ ^]^ It remains to be seen whether the same can be achieved with MMS, since this was not explored in studies where magnetic forces were applied to neurons in culture.^[^ ^36^, ^37^ ^]^ However, in this study, MMS did enable faster control of astrocytes than optogenetics, as the triggering of Ca^2+^ signals with MMS was on a sub‐second time scale (Figure 1c), while the rise time was in the range of several seconds with optogenetics.^[^ ^10^ ^]^ In addition, the elevation in ABP following the stimulation of VLM astrocytes also occurred faster with MMS (Figure 5) than with optogenetics.^[^ ^30^ ^]^ Another issue with MMS is that, in its current form, invasive intracranial injection is still required. Several strategies have been explored to enable systemically administered IONPs to cross the blood‐brain barrier,^[^ ^56^ ^]^ including focused ultrasound^[^ ^57^ ^]^ and magnetic hyperthermia.^[^ ^58^ ^]^ Therefore, it could be possible to eliminate intracranial injection and make MMS minimally invasive. Lastly, in the current implementation of MMS, relatively high concentrations of magnetic particles have been employed. Encouragingly, it has been shown that, after cultured astrocytes were exposed to a concentrated IONP dispersion containing 4 mm iron (about 2.5 mm iron in 0.25 mg mL^‐1^ SiMAG in this study), cell viability and glucose and glutathione metabolism were not compromised and reactive oxygen species production increased only transiently over a seven‐day period.^[^ ^49^ ^]^ There are also strategies to reduce the particle concentration needed for effective MMS. The saturation magnetization of the SiMAG particles, which contain maghemite cores, is only about 22% of that of the magnetite‐containing BioMag particles (Figure S3b, Supporting Information). By switching to magnetite‐containing particles, a larger force can be produced via particles of the same size, thus a reduced amount of particles would need to be attached to the cells. In addition, after small IONPs (15 nm) are sparsely attached to the cell membrane, it is possible to aggregate them into large ones (> 200 nm) by exposing the cells to a static magnetic field (0.15 T, 1 hour).^[^ ^59^ ^]^ This may allow the particle concentration to be further decreased.

In conclusion, here we report the development and validation of a novel method for remote activation of Ca^2+^ and ATP signaling responses in astrocytes, adding to the arsenal of cell control technologies that will help advance our understanding of brain function and combat CNS disorders.

## 4 Experimental Section

### Animal Care and Use

Cell cultures were prepared from the brain tissue of male and female Sprague‐Dawley rat pups (postnatal day 2–5). In vivo experiments were performed on young adult male Sprague‐Dawley rats (80–120 g, or 270–310 g). The rats were group‐housed and maintained on a 12‐h light cycle (lights on 07:00) and had ad libitum access to water and food. All animal procedures were approved by the UK Home Office (Project Licence No. PECE77103) and University College London's Animal Welfare and Ethical Review Body. The ARRIVE guidelines 2.0 are followed when reporting in vivo experiments. Sample size estimation and inclusion and exclusion criteria are described in the “Statistical analysis” section. No randomization or blinding was done.

### Cell Culture

Primary cultures of cortical astrocytes were prepared as described in detail previously.^[^ ^60^, ^61^ ^]^ After dissection and dissociation of the cortical tissue, the cells were plated on T75 culture flasks (Thermo Fisher Scientific) coated with poly‐*D*‐lysine (Merck Millipore). Cultures were maintained in a medium containing Dulbecco's Modified Eagle Medium (DMEM) with high glucose and GlutaMAX supplement, 10% fetal bovine serum (FBS), 100 U mL^−1^ penicillin and 100 µg mL^−1^ streptomycin (all from Gibco) at 37 °C in a humidified atmosphere of 5% CO_2_ and 95% air. After 7–8 days, the culture flasks were shaken on an orbital shaker at 180 rpm for 5–6 h to remove microglia and oligodendrocyte precursor cells. The remaining cells were re‐plated on 12 mm circular coverglasses (Gerhard Menzel GmbH) coated with poly‐*D*‐lysine. The coating was done by immersing each coverglass in a poly‐*D*‐lysine solution (300 µL, 25 µg mL^−1^) for 1 h. About 75000 cells were plated onto one coverglass. The cells were used between 10–21 days in vitro. Astrocyte cell cultures thus obtained have a high purity (Figure S1, Supporting Information).

Mixed neural cell cultures were prepared as described in detail previously.^[^ ^62^ ^]^ After dissection and dissociation of the cortical tissue, the cells were plated on poly‐*D*‐lysine‐coated 12 mm coverglasses and maintained in a medium containing Neurobasal‐A, 2% B‐27 supplement, 1% GlutaMAX supplement, 100 U mL^−1^ penicillin and 100 µg mL^−1^ streptomycin (all from Gibco) at 37 °C in a humidified atmosphere of 5% CO_2_ and 95% air. Cells were used between 7–14 days in vitro.

### Iron Oxide Particles

Five types of iron oxide particles were used in this study (Table S1, Supporting Information). Images of the particles were acquired with SEM and optical microscopy (Figure S3a, Supporting Information). A superconducting quantum interference device (SQUID) was used to measure the magnetic moment (***m***) of the particles at a series of magnetic field strengths (***H***). The mass of the particles was divided by their density to give volume (*V*), and ***m*** was divided by *V* to give particle magnetization (***M***). For the Fe_3_O_4_ particles (Figure 1d), the ***M***‐***H*** measurements were modeled by a sigmoidal curve by solving for the parameters of the function

(1) $$f\left(x\right)=\frac{b_{1}x}{\sqrt{b_{2}+b_{3}x^{2}}}$$

using the nonlinear regression model fitting function “fitnlm” in MatLab (MathWorks). For other types of particles (Figure S3b, Supporting Information), the ***M***‐***H*** measurements were modeled by a cubic spline curve using the “spline” function in MatLab.

### Ligand Coupling Methods

The iron oxide particles were functionalized with either collagen or anti‐GLAST. Collagen was used to promote the binding of magnetic particles to the cell membrane regardless of cell type. Anti‐GLAST is a monoclonal antibody that binds specifically to an extracellular epitope of the transmembrane protein GLAST that is expressed by astrocytes, Bergmann glia, Müller glia and radial glia, but not by neurons, oligodendrocytes, microglia, or neuronal progenitors.^[^ ^29^ ^]^

The Fe_3_O_4_ particles, which were in dry powder form, were coated with collagen through a procedure described previously.^[^ ^63^ ^]^ First, Fe_3_O_4_ particles (20 mg) were incubated with a mixture of type I collagen solution (50 µL, 3.0 mg mL^−1^, Sigma‐Aldrich), phosphate‐buffered saline (PBS, 200 µL) and NaOH solution (5 µL, 1 m) at 37 °C for 1 h (mass_ligand_ (µg)/mass_particle_ (mg) = 7.5). Then the particles were washed with PBS three times, resuspended in PBS (500 µL), and stored at 4 °C.

All other particles consisted of one or multiple iron oxide cores embedded in a matrix and carried surface carboxyl groups, enabling ligand coupling through a chemical reaction. The carboxyl groups, upon being activated by 1‐ethyl‐3‐(3‐dimethylaminopropyl) carbodiimide (EDAC), can react with amino groups in proteins to form covalent amide bonds.

Ligand coupling for BioMag Plus particles was performed according to the manufacturer's instructions. The particles (1 mg) were washed four times with a 2‐(*N*‐morpholino)ethanesulfonic acid (MES, Sigma‐Aldrich) buffer (50 mm, pH 5.2), followed by activation in the MES buffer (120 µL) containing 13.33 mg mL^−1^ EDAC (VWR International) for 30 min at room temperature in a rotator. Then, the particles were washed four times with the MES buffer, resuspended in a mixture of an anti‐GLAST solution (50 µL, 0.10 mg mL^−1^, Miltenyi Biotec) and the MES buffer (10 µL), and incubated for 16 h at room temperature in a rotator (mass_ligand_ (µg)/mass_particle_ (mg) = 5.0). After that, the particles were washed twice with the MES buffer and incubated in a glycine solution (1 m, pH 8.0) for 30 min at room temperature in a rotator. Lastly, the particles were washed four times with a storage solution containing tris(hydroxymethyl)aminomethane (Tris, 0.01 m), NaCl (0.15 m), bovine serum albumin (BSA, 1 mg mL^−1^), ethylenediaminetetraacetic acid (EDTA, 0.001 m), and sodium azide (1 mg mL^−1^), before being resuspended in the storage solution (100 µL) and stored at 4 °C.

The protocols for preparing anti‐GLAST‐coupled BioMag Maxi and SiMAG particles were optimized to enhance ligand coupling efficiency (Figures S6 and S7, Supporting Information, respectively). The finalized version for BioMag Maxi particle preparation is as follows. First, the particles (1 mg) were washed four times with a coupling buffer (pH 5.5) containing NaCl (0.15 m) and K_2_HPO_4_ (0.01 m), followed by activation in the coupling buffer (120 µL) containing 33.33 mg mL^−1^ EDAC for 10 min at room temperature in a rotator. Then, the particles were washed twice with the coupling buffer, resuspended in a mixture of the anti‐GLAST solution (50 µL) and the coupling buffer (10 µL), and incubated for 1 h at room temperature in a rotator (mass_ligand_ (µg)/mass_particle_ (mg) = 5.0). Lastly, the particles were washed four times with PBS, resuspended in PBS (100 µL), and stored at 4 °C. The protocol for SiMAG particles was similar, except that the coupling buffer was an MES buffer (100 mm, pH 5.0), the concentration of the EDAC solution for particle activation was 4.17 mg mL^−1^, and activated particles were incubated with the anti‐GLAST solution for 2 h.

To coat BioMag Plus, BioMag Maxi or SiMAG particles with collagen, the particles (1 mg) were first washed twice with the appropriate wash buffer. Then the particles were resuspended in a mixture of the collagen solution (16.7 µL) and the wash buffer (103.3 µL), and incubated for 1–2 h at room temperature in a rotator (mass_ligand_ (µg)/mass_particle_ (mg) = 50.0). Lastly, the particles were washed three times with PBS, resuspended in PBS (100 µL), and stored at 4 °C.

### Ligand Coupling Efficiency

Ligand coupling efficiency was determined by measuring how much of the ligand remained in the solution after the coupling process.

The concentration of collagen was quantified using the Bradford protein assay. The Coomassie (Bradford) Protein Assay (Thermo Scientific) reagent was modified to contain 0.035 mg mL^−1^ sodium docecyl sulphate (SDS),^[^ ^64^ ^]^ in order to increase the steepness of the collagen standard curve and help resolve smaller differences. First, the test solution (5 µL) was added to the assay reagent (250 µL), and the mixture was vortexed and left at room temperature for 10 min. Then, the mixture was vortexed again, before a portion of it (100 µL) was taken out and added to a 96‐well plate. Finally, the absorbance was measured at 570 nm using a microplate reader (Multiskan FC, Thermo Scientific).

The anti‐GLAST antibody used in this study was tagged with either phycoerythrin (PE) or allophycocyanin (APC). For ligand coupling efficiency measurements, anti‐GLAST‐PE was used, and its concentration was estimated by measuring the absorbance at 560 nm using a microplate reader (Varioskan LUX, Thermo Scientific) and comparing the reading to a standard curve.

### Magnets

In order to accurately determine the mechanosensory threshold of astrocytes in culture, an electromagnet that could exert a uniform and tunable force on magnetic particles within a large area was designed based on the well‐known Faraday susceptibility measuring method^[^ ^65^ ^]^ and the report by Garber and colleagues.^[^ ^66^ ^]^ This device, named the “yoke” magnet, has two opposing pole pieces with a reciprocal parabolic profile. The shape of the pole pieces was determined as follows.

When magnetic particles in a dilute suspension are subjected to a moderate magnetic field such that the induced magnetization in the particles is not saturated and increases approximately linearly with the applied field, the force they experience can be calculated using the following equation^[^ ^67^ ^]^

(2) $$F_{m}=\frac{V\Delta \chi }{\mu_{0}}\;\left(B\cdot \nabla \right)B$$

where *V* is the volume of the particle, Δ*χ* = *χ*_m_ − *χ*_f_ is the difference between the volumetric magnetic susceptibility of the particle (*χ_m_*) and that of the suspending fluid (*χ_f_*), *μ_0_* is the permeability of free space (4*π* × 10^−7^ N A^−2^), and ***B*** is the magnetic flux density field. According to this equation and Garber and colleagues,^[^ ^66^ ^]^ having a uniform magnetic force on a given type of particles in the electromagnet pole gap means that the magnetic field must fulfil the following condition

(3) $$-B_{y}\frac{dB_{x}}{dx}+B_{x}\;\frac{dB_{x}}{dy}=\;\mathrm{constant}$$

As an approximation, the variations with respect to *x* are ignored and the condition is simplified to

(4) $$B_{x}\;\frac{dB_{x}}{dy}=\;\mathrm{constant}$$

This yields

(5) $$B_{x}=\sqrt{ay+b}$$

where *a* and *b* are constants. Again following Garber and colleagues,^[^ ^66^ ^]^ *B_x_* in the gap is assumed to be inversely proportional to the gap width

(6) $$B_{x}\propto \frac{1}{\Delta x}$$

with Δ*x* being half the gap width at level *y*. It follows that

(7) $$\Delta x\;=\sqrt{\frac{c}{ay+b}}$$

where *c* is another constant. This formula determines the surface shape of the pole heads for positive values of *y*. For negative values of *y* the geometry of the pole heads is given by

(8) $$x\;=\;-y+d_{min}$$

where *d_min_* is half the minimum separation of the poles (at *y* = 0), that is

(9) $$d_{min}=\sqrt{c/b}$$

As a balance between maximizing *B_x_* and allowing a large enough pole gap, the values eventually chosen for the constants defining the shape of the poles in mm were: *a* = −0.1, *b* = 4 and *c* = 100.

This design was subjected to finite element analysis using the Vector Fields Opera‐3D v12 simulation software (Cobham Technical Services), and the simulation showed that, within a large portion of the pole gap, the magnitude and direction of (***B*** · ∇) ***B***, which is a good correlate of the force exerted on magnetic particles, would be highly uniform. To make the magnet, a soft SiFe alloy (silicon core iron “B‐FM”, Carpenter Technology Corporation) was fashioned into the pole pieces, which were linked together by a SiFe alloy cylinder (20 mm in diameter), and then a copper wire was wound around the cylinder to produce the solenoid coil (1035 turns in 10 layers). To enable live cell imaging, a culture chamber that can hold a 12 mm coverglass at a defined position with respect to the pole pieces was made using a Formiga P100 plastic laser‐sintering 3D printer (EOS GmbH). To verify the designed feature of the yoke magnet, a GM08 Gauss meter (Hirst Magnetic Instruments) was used to measure the magnetic flux density at a series of points along the midline between the pole pieces at either 10 mm or 2 mm above the base of the magnet across a range of input current amplitudes. A close match between simulated and measured values was found (Figure S2b, Supporting Information), thus validating the simulations (Figure S2a, Supporting Information). When the cell culture is placed at 10 mm above the magnet base, namely, the midpoint of the magnet's height, the forces exerted on magnetic particles are entirely horizontal and parallel to the imaging plane, but at 2 mm above the magnet base, the forces have appreciable vertical components (Figure S2e, Supporting Information).

To perform magnetomechanical stimulation of astrocytes in vivo, a device named “Magnetic Mangle” was made based on the design proposed by Cugat and colleagues.^[^ ^38^ ^]^ It consisted of four ring‐shaped, diametrically magnetized, N42 grade NdFeB permanent magnets (outer diameter = 20 mm, inner diameter = 6 mm, height = 20 mm, Magnet Expert Ltd) arranged on a platform in a rectangular grid. The magnetic field in the inter‐magnet space could be drastically changed by changing the directions that the poles of the magnets pointed towards. The distances between the magnets themselves could also be adjusted. The platform was mounted on a track, so that the magnetic field could be applied or terminated by moving the magnets into or away from the target location. Similar to the yoke magnet, the direction of the force produced on magnetic particles by the Magnetic Mangle was largely parallel to the xy plane as defined in Figure 3c. The magnetic field in the inter‐magnet space was modeled using the Opera simulation software and the values were verified by comparing them to measurements obtained using the Gauss meter.

Finally, a 9.4 T VNMRS horizontal bore magnetic resonance imaging system (Agilent Technologies) was used as a magnetic actuation device. The edge of the magnet bore and a position 0.66 m away from the edge were chosen as “on” and “off” locations, respectively. At each location, magnetic field was measured at 125 points of a cubic lattice, whose edge was 20 mm long and contains 5 evenly spaced points.

### Force and Stress Calculation

The magnetic force on a point‐like magnetic dipole ***m*** in a magnetic flux density field ***B*** is defined as^[^ ^67^, ^68^ ^]^

(10) $$F_{m}=\left(m\cdot \nabla \right)\;B$$

In case of a magnetic particle suspended in a weakly diamagnetic medium such as water, the total magnetic moment on the particle can be expressed as

(11) m=VM

where *V* is the volume of the particle and ***M*** is its volumetric magnetization. Therefore

(12) $$F_{m}=\;V\left(M\cdot \nabla \right)B$$

This can be expanded to

(13) $$\begin{array}{ccc} F_{m} & = & V\left[M_{x}\frac{\partial B_{x}}{\partial x}+M_{y}\frac{\partial B_{x}}{\partial y}+M_{z}\frac{\partial B_{x}}{\partial z},\;M_{x}\frac{\partial B_{y}}{\partial x}+M_{y}\frac{\partial B_{y}}{\partial y}+M_{z}\frac{\partial B_{y}}{\partial z},\right.\\ & & \left.M_{x}\frac{\partial B_{z}}{\partial x}+M_{y}\frac{\partial B_{z}}{\partial y}+M_{z}\frac{\partial B_{z}}{\partial z}\right] \end{array}$$

The ***B*** fields of the bespoke magnets were estimated by finite element analysis and verified by measurements. The ***B*** field of the MRI scanner was measured. The magnetization ***M*** of a particular type of particles upon exposure to a given magnetic field strength ***H*** was obtained from the curve fitted onto the relevant ***M***‐***H*** measurements. ***H*** is related to ***B*** by the following equation

(14) $$B\;=\mu_{0}\;\mu_{r}H$$

where *μ_r_* is the relative permeability of the medium. The value of *μ_r_* for either air (1.000000,37) or water (0.999990,93) is very close to 1. Therefore, for a given ***B*** field, the corresponding ***H*** field and in turn, the magnetization ***M*** of the particles could be calculated.

With all these quantities known, force per unit volume was estimated for a particular type of particles actuated by a particular magnet. At midpoint along the height of the yoke magnet (10 mm above base), the force on particles of the same type and size within the cell culture location was highly uniform (Figure S2e, Supporting Information). However, as the pole gap was too narrow for a typical microscope lens to image the plane at 10 mm above base, the cell culture was placed at 2 mm above base for calcium imaging experiments. At this height, the force was more variable with location, but the inhomogeneity was much less severe around the midline (within the white ellipse in Figure S2e in the Supporting Information), therefore calcium imaging was restricted to this region. After the minimum input current required to trigger a Ca^2+^ signal in an astrocyte was determined, the force applied was deemed to be the median of the force per unit volume values estimated for that input current and bounded by the central ellipse.

To estimate particle volume, SiMAG and fluidMAG particles were assumed to be monodisperse spheres with diameters equal to the nominal sizes given by the manufacturers (Table S1, Supporting Information), while BioMag Plus particles were assumed to be discs with a diameter of 1.5 µm and a height of 0.2 µm. Because the shape and size of collagen‐coated Fe_3_O_4_ particle clusters were highly irregular, their volumes were individually estimated as follows. First, the working concentration and volume of the Fe_3_O_4_ suspension to incubate cell cultures with were established to be 0.22 mg mL^−1^ and 353 µL, respectively, in a well of a 24‐well plate. This produced a sparse distribution of particle clusters, and cells were mostly associated with single, distinct ones. Then, in bright‐field micrographs, the Fe_3_O_4_ clusters attached to cells were located, and their base areas calculated using ImageJ. Finally, to be able to estimate the volume of a Fe_3_O_4_ cluster from its base area, the relationship between the two was examined using SEM. Three astrocyte cultures adorned with Fe_3_O_4_ particles were imaged (see below for SEM protocol). For each field of view (FOV), two images were taken at 10° and 20° tilt of the stage respectively. Using MountainsMap SEM (Digital Surf sarl), stereoscopic 3D surface reconstruction was performed on each pair of images, followed by tilt correction, producing a topographic map. Then regions of interest (ROIs) were drawn and the base area and volume of each Fe_3_O_4_ cluster were calculated. From this data, the relationship between base area and volume was inferred and used to estimate the volume of the Fe_3_O_4_ clusters associated with cells.

After the force exerted on a magnetic particle was determined, the stress it would produce at the point of contact with a cell was calculated. Stress was equal to the force acting over the cross‐sectional area of an object divided by the cross‐sectional area. For SiMAG and fluidMAG particles, the area was assumed to be the cross‐sectional area through the centre of the sphere. For BioMag Plus particles, the area was assumed to be the area of the disc face. For Fe_3_O_4_ particles, the base areas of the clusters were used.

### Calcium Imaging

Cell cultures were washed twice with Hanks' Balanced Salt Solution (HBSS) before being incubated in HBSS containing iron oxide particles for 0.5–1 h at room temperature. Then the cells were washed twice with HBSS and incubated in HBSS containing Fura‐2 AM (4 µm, Invitrogen) and Pluronic F‐127 (0.04%, Invitrogen) for 0.5–1 h at room temperature in the dark, followed by another two washes with HBSS. Changes in individual cells’ [Ca^2+^]_i_ during the MMS experiments were monitored using an Olympus IX71 inverted microscope with an Andor CCD camera. Excitation light was provided by a xenon arc lamp with the beam passing through a monochromator at 340 and 380 nm (Cairn Research) and emitted fluorescence at 515 nm was registered.

To estimate the mechanosensory threshold of astrocytes, recordings from the experiments that utilized the yoke magnet and collagen‐coated Fe_3_O_4_ particles were analyzed as follows. The ratio between Fura‐2 fluorescence excited at 340 nm and that at 380 nm, which gives an accurate indication of changes in [Ca^2+^]_i_, was first calculated. A cell was considered responsive if the maximum value reached by the Fura‐2 ratiometric signal during the 20 s following the start of the stimulus was greater than the baseline (calculated as the mean over the 20 s prior to stimulation) by more than 25%.^[^ ^69^ ^]^ Cells were subjected to a series of magnetomechanical stimuli of increasing magnitudes, and for a given cell, the first stimulus that triggered a response was considered to be the threshold. The distribution of the threshold values obtained from different cells was modeled by a lognormal curve by solving for the parameters *A*, *μ* and *σ* of the function

(15) $$f\;\left(x\right)=\frac{A}{x\sigma \sqrt{2\pi }}\;\exp\left(\frac{-{\left(\ln x-\mu \right)}^{2}}{2\sigma^{2}}\right)$$

using the “fitnlm” function in MatLab.

### Measurement of ATP Release

Cell cultures grown on 12 mm coverglasses were transferred to custom‐made cups (inner diameter = 13 mm, wall thickness = 0.5 mm) and rested in culture medium for 1 h in the incubator. This was followed by two washes with HBSS and incubation with HBSS (265 µL) containing iron oxide particles for 1 h at room temperature. At the beginning of the experiment, a pre‐stimulation sample (80 µL) was drawn and immediately frozen on dry ice. Then the experimental manipulation was carried out and a post‐stimulation sample (80 µL) was collected and frozen on dry ice.

ATP concentration in the cell culture medium samples was measured using an assay (CellTiter‐Glo, Promega) based on the luciferin‐luciferase reaction. Specifically, each sample (20 µL) as well as a series of ATP standard solutions (20 µL, 0–80 nm) were added to an opaque 384‐well plate (Greiner Bio One International), and each of them was mixed with the luciferin‐luciferase reagent (20 µL). The bioluminescence was recorded using an IVIS Lumina imaging system (PerkinElmer), and the photon count was converted to ATP concentration using the standard curve.

### Stereotaxic Delivery of Magnetic Particles

The rats were anesthetized with an intraperitoneal (i.p.) injection of a mixture of ketamine (75 mg per kg of body weight) and medetomidine (0.5 mg per kg of body weight). After the head of the animal was placed in a stereotaxic frame (David Kopf Instruments), a midline incision on the scalp was made and small cranial holes were drilled, allowing magnetic particles to be injected into the brainstem using a microinjection syringe (5 µL, Model 75 RN, Hamilton Company) and needle (26s gauge, Small Hub RN, 43 mm, point style AS, Hamilton Company) at a rate of 0.05 µL min^−1^. The coordinates for the injections (Table S9, Supporting Information) were selected according to the stereotaxic atlas of the rat brain^[^ ^70^ ^]^ and a previous study.^[^ ^30^ ^]^ The coordinates used in small rats (80–120 g) were experimentally determined. After the particles were delivered, the wound was sutured and the animal was given atipamezole (1 mg per kg of body weight, i.p.) to reverse anaesthesia and buprenorphine (0.03 mg per kg of body weight, i.p.) for pain relief.

### Magnetic Resonance Imaging

To determine the location and fate of the magnetic particles once they were injected into the brainstem of rats, MRI was performed on the 9.4 T MRI scanner with a 72 mm inner diameter volume coil (RAPID Biomedical) for radiofrequency transmission and a 4‐channel array head coil (RAPID Biomedical) for signal reception. Anesthesia was induced with 4% isoflurane and maintained with 1.5% isoflurane. Body temperature was maintained with a heated waterbed. Respiratory rate and body temperature were monitored with a pneumatic pillow sensor and a rectal thermister probe respectively, both connected to a MR‐compatible monitoring and gating system (SA Instruments). A T_2_*‐weighted gradient‐echo sequence was used: TE = 6.5 ms, TR = 2230 ms, flip angle = 56°, number of averages = 5, FOV = 28.8 mm × 28.8 mm, matrix = 192 × 192, slice thickness = 0.15 mm, interslice distance = 0 mm. The images have an isotropic resolution of 150 µm.

Affine registration of the MR images was performed using the NifTK software.^[^ ^71^ ^]^

### Immunofluorescence

Cell cultures were fixed with a buffered 4% paraformaldehyde (PFA) solution (VWR Chemicals) for 10 min at room temperature, washed twice with PBS, and incubated with a blocking buffer (PBS with 5% donkey serum, 0.3% Triton X‐100, all from Sigma‐Aldrich) for 1 h at room temperature. After removing the blocking buffer, each culture was incubated with a dilution buffer (PBS with 10 mg mL^−1^ BSA, 1% donkey serum, 0.3% Triton X‐100, and 0.1 mg mL^−1^ sodium azide) containing one or more primary antibodies for 1 h at room temperature. Next, each culture was washed three times with PBS and incubated with the dilution buffer containing one or more secondary antibodies for 1 h at room temperature. After staining was done, each culture was washed three times with PBS and incubated with a 4’,6‐diamidino‐2‐phenylindole (DAPI) solution (PBS with 2.86 µm of DAPI) for 5 min. Lastly, the samples were mounted onto microscope slides using a histology mounting medium (Fluoroshield, Sigma‐Aldrich).

To prepare brain sections, the animals were anesthetized with sodium pentobarbital (60 mg per kg of body weight, i.p.) and underwent transcardial perfusion with saline and the buffered 4% PFA solution. After the brain was dissected out, it was immersed in a buffered 2% PFA solution overnight, washed twice with PBS, and immersed in PBS with 30% sucrose (Sigma‐Aldrich). The brainstem was then dissected out and sectioned (16 µm). The sections were mounted onto gelatin‐coated 12 mm coverglasses. The immunostaining protocol for brainstem sections was the same as that for cell cultures, except that incubation with primary antibodies consisted of 1 h at room temperature followed by 24 h at 4 °C.

Immunofluorescence images of cell cultures were acquired with a Zeiss Axio Observer Z1 inverted microscope. Immunofluorescence images of brainstem sections were acquired with a Zeiss LSM 880 confocal microscope.

### Scanning Electron Microscopy

Cell cultures were fixed with a buffer containing 0.1 m sodium cacodylate, 2% paraformaldehyde and 1.5% glutaraldehyde (pH 7.3, all from Sigma‐Aldrich) overnight at 4 °C, and postfixed with a buffer containing 0.1 m sodium cacodylate and 1% osmium tetroxide (Sigma‐Aldrich) at 4 °C for 30 min. Then the cells were washed with a sodium cacodylate buffer (0.1 m), rinsed with distilled water, dehydrated in ethanol, and dried using CO_2_. After that, the specimens were mounted on aluminum stubs using carbon adhesive tabs and coated with a thin layer of gold/palladium or carbon using an ion beam coater (Gatan). For each culture, images were acquired at 700 times magnification with a JEOL JSM‐7401F field emission scanning electron microscope (JEOL Ltd.) at four positions, one in every quadrant.

### Magnetomechanical Stimulation of Astrocytes In Vivo

For the experiments that used the MRI scanner as the magnetic device, the animals were first anesthetized with isoflurane (4% for induction, 1.5% for maintenance) and the femoral vein and artery were cannulated to gain vascular access. Urethane (Sigma‐Aldrich, 1.3 g per kg of body weight) was administered intravenously (i.v.) and isoflurane was discontinued. Adequate anesthesia was ensured by maintaining stable levels of ABP and heart rate showing lack of responses to a paw pinch. The body temperature was maintained with a heated waterbed at 37.0 ± 0.5 °C. ABP, body temperature and respiratory rate were recorded with the physiological monitoring system for the MRI scanner. For baseline recordings, the animal was kept at the “off” position which was 0.66 m away from the edge of the scanner bore. To apply the fringe magnetic field, the animal was moved to the “on” position where its head was at the edge of the scanner bore.

For the experiments that used the Magnetic Mangle, the animals were anesthetized with urethane (induction: 1.3 g per kg of body weight, i.p.; maintenance: 10–25 mg per kg of body weight per hour, i.v.). The femoral artery and vein were cannulated for the measurements of ABP and administration of anesthetic, respectively. The body temperature was maintained at 37.0 ± 0.5 °C. MMS of brainstem astrocytes was achieved by moving the Magnetic Mangle so that the magnets surrounded the head of the animal.

To calculate MAP from the ABP signal, the wave within each cardiac cycle was integrated and then divided by the duration of the cycle. Heart rate was derived from the ABP signal by calculating the frequency of cardiac cycles.

### Statistical Analysis

Since no previous study of MMS‐induced change in blood pressure had been conducted, interim sample size estimation was performed after initial in vivo experiments. A naive group and an experimental group (injection of a 0.5 mg/mL suspension, POD1) with a sample size of 6 measurements in each group yielded an effect size of 3.0 (Cohen's *d*). Based on these results, a minimum sample size of 3 was required for a two‐tailed two‐sample *t*‐test to have a power of 0.9 at the significance level of 0.05. Therefore a sample size of 6 and 5 was used for experiments with the MRI scanner and the Magnetic Mangle, respectively. Animals were excluded if the injection location was off target, or if no stable baseline could be established during blood pressure recording.

Measurements of ATP release from astrocyte cultures and changes in blood pressure and heart rate as a result of in vivo MMS were subjected to statistical analysis.

1. Pre‐processing of data. For ATP release measurements, change in [ATP]_e_ was calculated by subtracting the initial [ATP]_e_ from the [ATP]_e_ after the experimental manipulation. For in vivo MMS experiments, change in MAP (*Δ*MAP) was calculated by subtracting the mean MAP during the pre‐stimulation period from the mean MAP during the stimulation period, and change in heart rate was calculated by subtracting the mean heart rate during the pre‐stimulation period from the mean heart rate during the stimulation period.
2. Data are presented as mean ± S.D. except in Figure 3i, where mean ± standard error is used.
3. Sample size is given in the relevant figure legends and supplementary tables.
4. Statistical methods. Two‐tailed Student's *t*‐test (*α* = 0.05) was used to test the null hypothesis that the mean value is equal to 0 or the null hypothesis that the difference between the means of two samples is 0. Linear regression analysis (*α* = 0.01) was used to determine the effects of one or multiple explanatory variables on the response variable.
5. Software. MatLab was used to conduct all statistical analysis.

## Conflict of Interest

The authors declare no conflict of interest.

## Supporting information

Supporting Information

## Data Availability

The data that support the findings of this study are available from the corresponding author upon reasonable request.
