# Supplementary material for: Remote and Selective Control of Astrocytes by Magnetomechanical Stimulation
Source: Adv Sci (Weinh). 2021 Dec 19;9(6):2104194. doi: 10.1002/advs.202104194 (PMC8867145; doi:10.1002/advs.202104194)
Supplement: Supplementary file 1 — Supporting Information [file ADVS-9-2104194-s001.pdf]

ADVANCED SCIENCE
Open Access

Supporting Information

for *Adv. Sci.,* DOI: 10.1002/advs.202104194

Remote and Selective Control of Astrocytes by Magnetomechanical Stimulation

*Yichao Yu, Christopher Payne, Nephtali Marina, Alla Korsak, Paul Southern, Ana García-Prieto, Isabel N. Christie, Rebecca R. Baker, Elizabeth M. C. Fisher, Jack A. Wells, Tammy L. Kalber, Quentin A. Pankhurst, Alexander V. Gourine, Mark F. Lythgoe**

Supporting Information

Remote and Selective Control of Astrocytes by Magnetomechanical Stimulation

Yichao Yu, Christopher Payne, Nephtali Marina, Alla Korsak, Paul Southern, Ana García-Prieto, Isabel N. Christie, Rebecca R. Baker, Elizabeth M. C. Fisher, Jack A. Wells, Tammy L. Kalber, Quentin A. Pankhurst, Alexander V. Gourine, Mark F. Lythgoe*

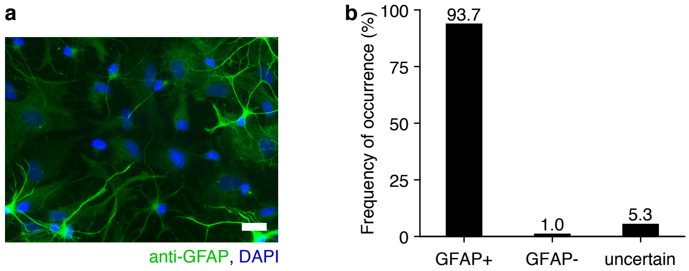

Figure S1. Characterization of astrocyte cultures.

a) Labeling of the glial fibrillary acidic protein (GFAP) by an anti-GFAP antibody and staining of the cell nuclei by 4',6-diamidino-2-phenylindole (DAPI) in an astrocyte culture. Scale bar = 20 µm. b) Purity of astrocyte cultures was determined using images such as the one in Panel a. A total of 1,338 cells were counted from 11 cultures derived from 4 rats.

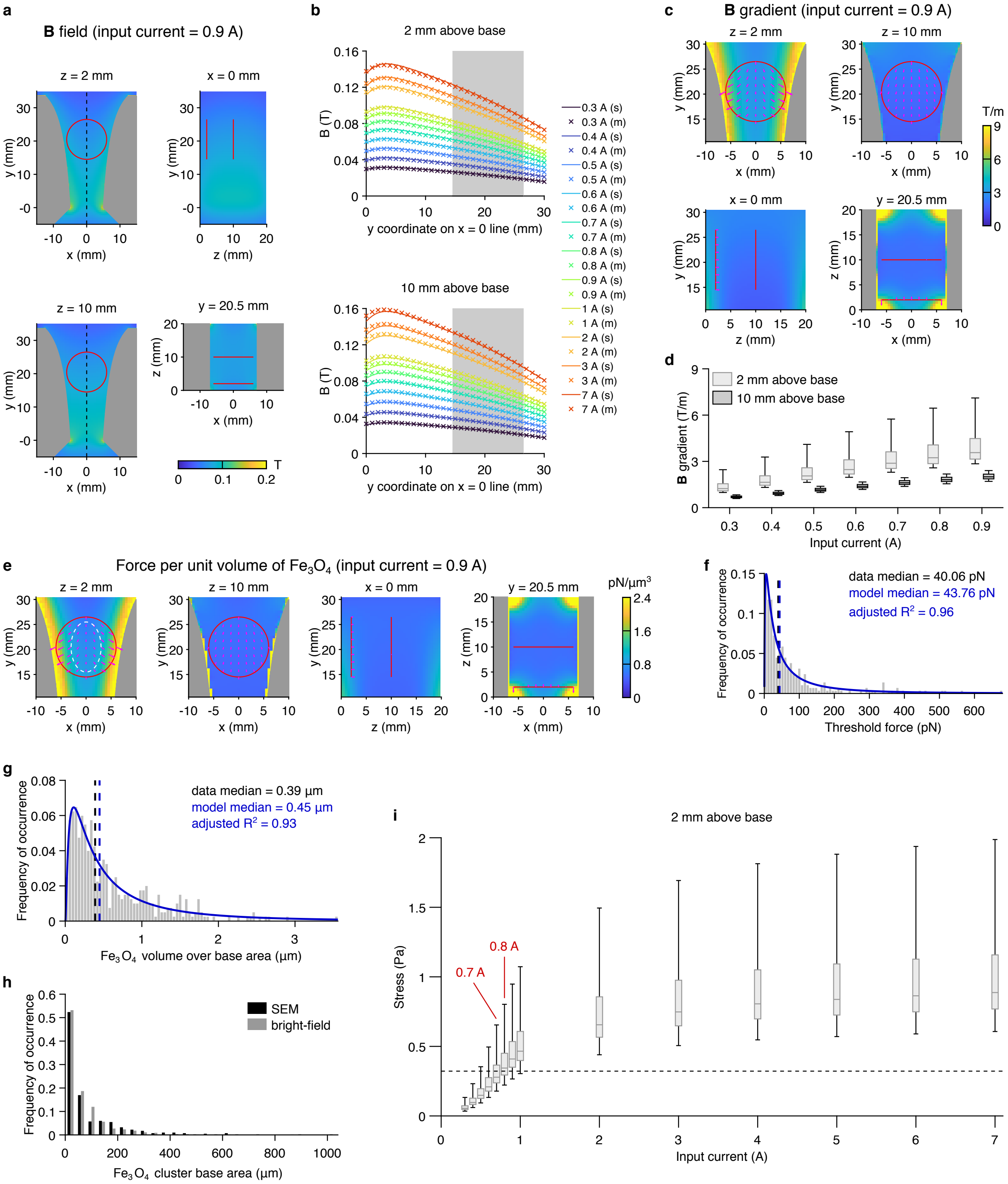

Figure S2. Additional results from investigations of astroglial mechanosensory threshold. a) Maps showing simulated values of magnetic flux density between the pole pieces of the yoke magnet. b) Comparison between simulated (s) and measured (m) magnetic flux density values along the x = 0 line (black dashed line in Panel a). Gray shade indicates the portion of the line that is within the cell culture location. c,d) Maps and summary statistics of the magnetic field gradient simulations for the yoke magnet. e) Maps showing the estimated values of force per unit volume of magnetite (Fe_3O_4) particles. In Panel (a,c,e): the red circles or lines indicate cell culture location; the magenta arrows indicate the directions and relative

magnitudes of the vectors; the white dashed ellipse demarcates the region of high uniformity at 2 mm above magnet base. f) The minimum forces required to trigger Ca^{2+} signals in 290 astrocytes. The data exhibit a lognormal distribution. g) Scanning electron microscopy (SEM) images of astrocyte cultures were analysed to determine the base area and volume of the Fe_3O_4 clusters attached to the cells (Figure 1f). Dividing the cluster volumes by the corresponding base areas gives the "Fe_3O_4 volume over base area" values, which display a lognormal distribution. n = 401 clusters. h) Fe_3O_4 cluster base areas obtained from SEM images have a similar distribution as those obtained from bright-field images. n = 551 clusters from bright-field images. i) Summary statistics of stress via Fe_3O_4 particles within the cell culture location at different input current amplitudes. To calculate this, the force per unit volume values (Panel e) were multiplied by the median of the lognormal model fitted to the Fe_3O_4 volume over base area values (Panel g). Blacked dashed line = 0.32 Pa. n = 441 points for each condition. In Panel (d,i): bar, median; box, quartiles; whiskers, range.

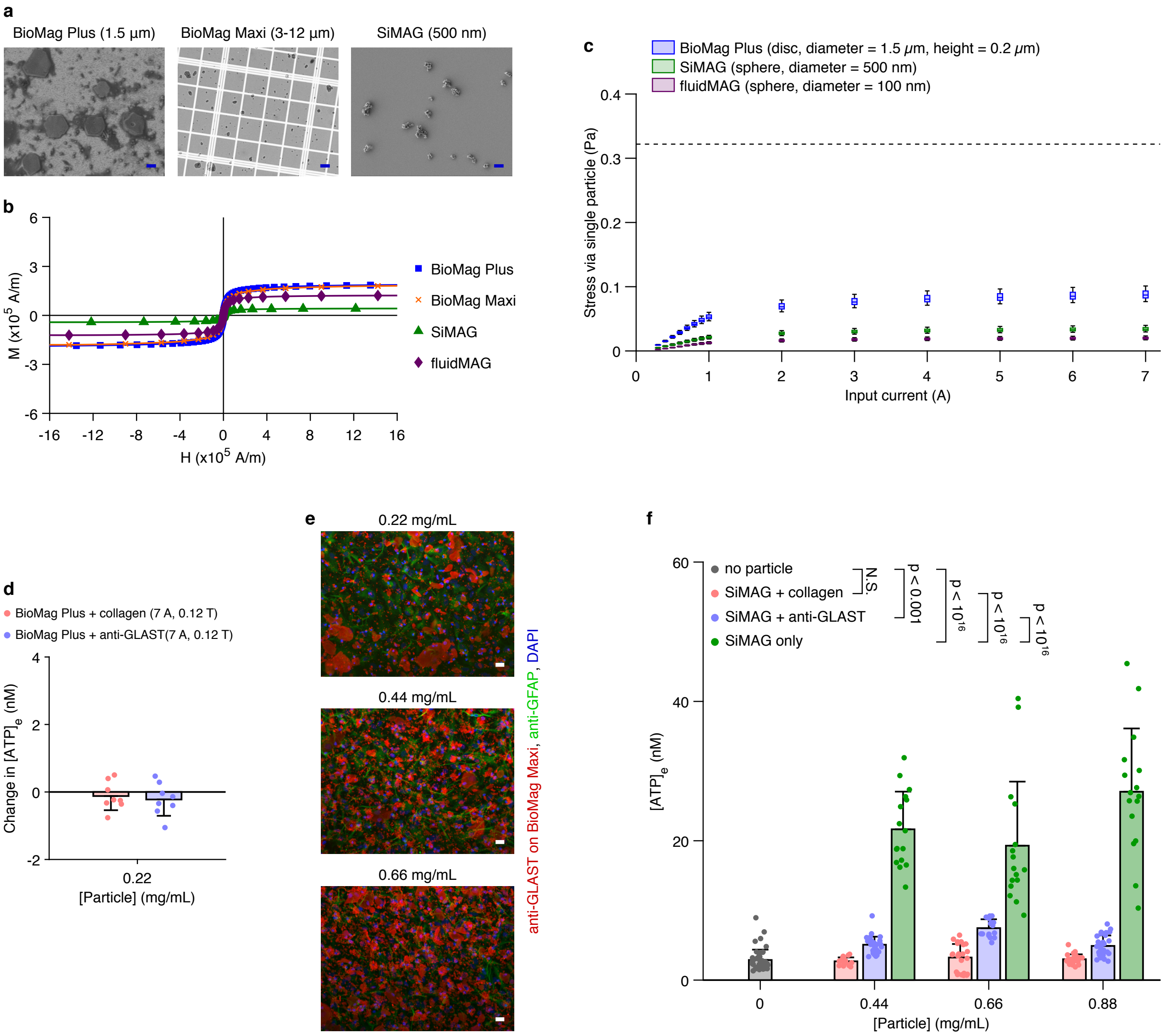

Figure S3. Additional results from particle evaluation experiments.

a) Scanning electron micrographs of BioMag Plus and SiMAG particles, and optical micrograph of BioMag Maxi particles. b) Particle magnetization measurements (***M***) at different magnetic field strengths (***H***), as well as the fitted curves. c) Estimation of the stresses generated by the yoke magnet through single particles within the cell culture location at 10 mm above the magnet base. $n = 441$ points for each condition. Bar, median. Box, quartiles. Whiskers, range. d) No change in extracellular adenosine triphosphate (ATP) concentration ($[ATP]_e$) following magnetomechanical stimulation (MMS) of cultured astrocytes by actuating BioMag Plus particles attached to the cells with the yoke magnet (7 A, 10 mm above base, median magnetic flux density = 0.12 T). $n = 8$ measurements for each condition. e) When BioMag Maxi particles functionalized with an antibody against the glutamate-aspartate transporter (GLAST) were applied to astrocyte cultures, the degree of particle aggregation increased as the concentration of the particle suspension increased. Scale bar = 20 µm. f) After 1 h of incubation with SiMAG particles, $[ATP]_e$ was drastically

different depending on the presence or absence of ligand on the surface of the particles. In comparison to the no particle condition: collagen-coated SiMAG particles had no effect on $[ATP]_e$ ($p = 0.869$); the use of anti-GLAST-coupled SiMAG particles led to appreciable elevations in $[ATP]_e$ ($p < 0.001$); and unmodified SiMAG particles caused $[ATP]_e$ to rise conspicuously ($p < 10^{-16}$). See Table S5. This phenomenon was likely due to the negative charge of the carboxyl groups on the surface of the SiMAG particles. When making collagen-coated particles, the ratio between ligand mass in µg and particle mass in mg was 50.0, resulting in complete masking of the carboxyl groups, whereas for anti-GLAST-coupled ones, the ratio was only 5.0, resulting in partial coverage of the particle surface. From left to right, n = 48, 16, 32, 16, 24, 16, 16, 24, 32 and 16 measurements. In Panel (d,f): data shown as mean ± standard deviation (S.D.).

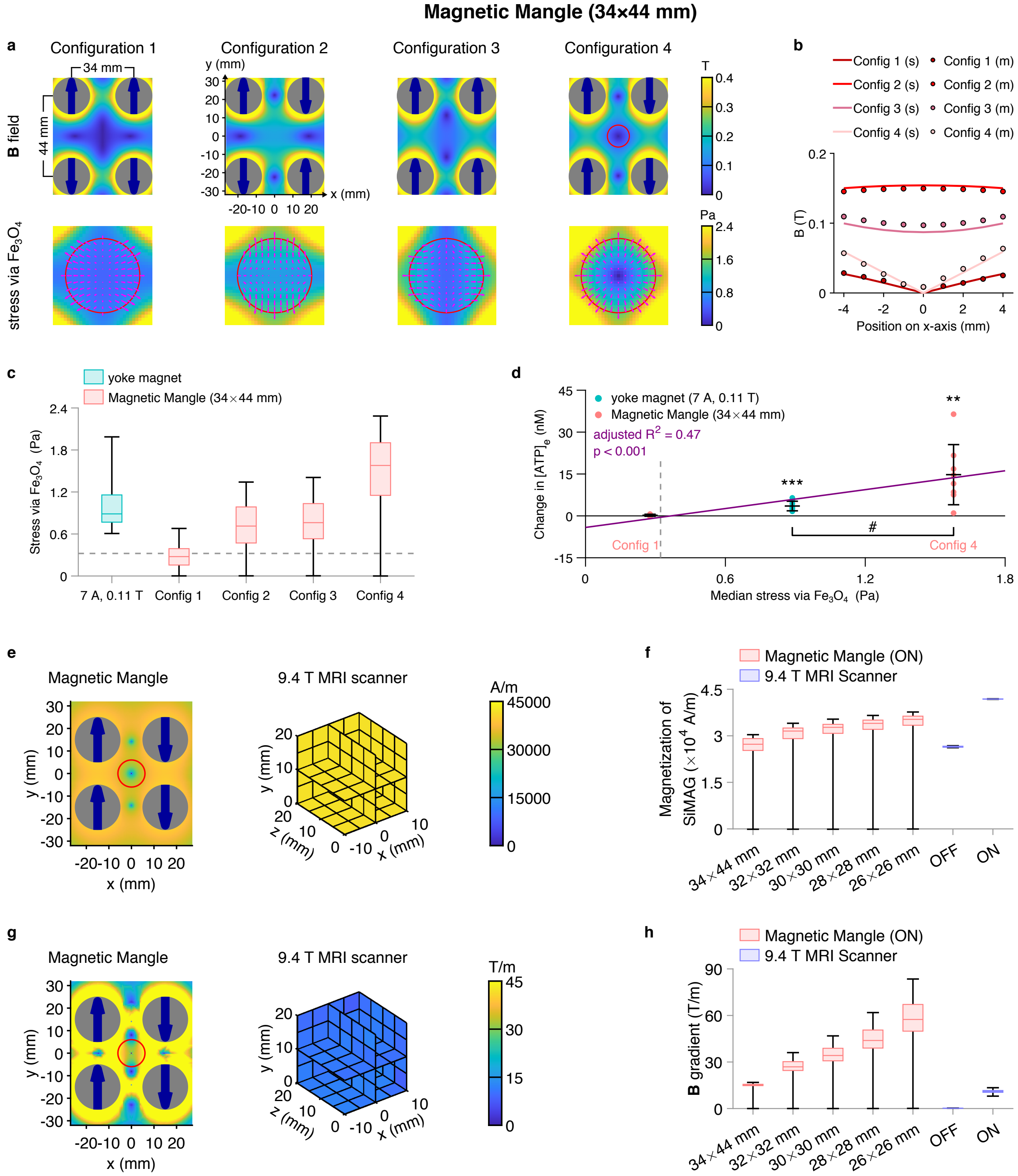

Figure S4. Additional results of magnetic device characterizations.

a) Top row: simulations of the magnetic field generated by the Magnetic Mangle when different configurations of magnet orientations were employed. Bottom row: estimated stress via Fe_3O_4 particles. Gray circles represent magnets. Indigo arrows indicate magnetization directions. b) Magnetic field measurements (m) matched well with simulated values (s). c) Summary statistics of the estimated stress via Fe_3O_4 particles produced by different configurations of the Magnetic Mangle. Configuration 4 produces the highest stress. d) In vitro MMS of astrocytes adorned with collagen-coated Fe_3O_4 particles showed that the

estimated stress output of the device predicted the $[ATP]_e$ changes. Purple line, linear regression (Table S6). Data shown as mean ± S.D.; *n* = 8 measurements for each condition; **, $p < 0.01$, two-tailed *t*-test of mean $[ATP]_e$ change equaling zero; ***, $p < 0.001$, same test; #, $p < 0.05$, two-sample two-tailed *t*-test. e,f) Maps and summary statistics of SiMAG magnetization values estimated for the Magnetic Mangle and the MRI scanner fringe field. g,h) Maps and summary statistics of magnetic field gradient values estimated for the Magnetic Mangle and the MRI scanner fringe field. In Panel (a,e,g): red circles indicate the cell culture location; magenta arrows indicate the directions and relative magnitudes of the vectors. In Panel (c,f,h): *n* = 441 points for each condition except "9.4 T MRI Scanner" where *n* = 125 points; bar, median; box, quartiles; whiskers, range.

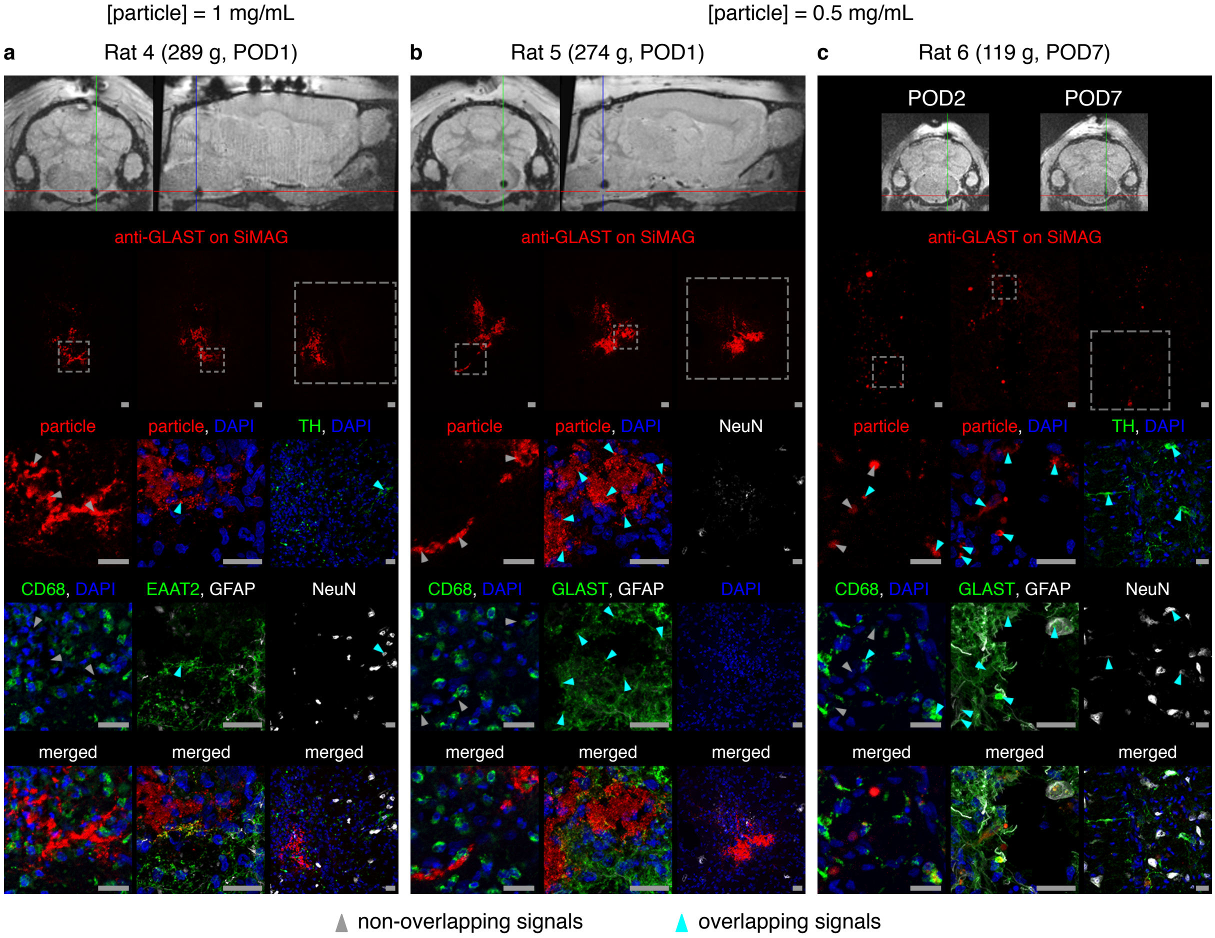

Figure S5. Additional results from investigations of SiMAG particle in vivo fate. Each rat received a unilateral 1 μL injection of an anti-GLAST-coupled SiMAG particle suspension. MRI was performed and the acquired images were affinely registered to each other. The cross hairs in MR images mark the same anatomical location in each brain. Animals were sacrificed at different time points and brainstem sections were stained for the microglial marker cluster of differentiation 68 (CD68), the astroglial markers GFAP, GLAST and excitatory amino acid transporter 2 (EAAT2), and the neuronal markers neuronal nuclei (NeuN) and tyrosine hydroxylase (TH). Three consecutive sections are shown for each rat. POD, post-operative day. Number of animals used = 3.

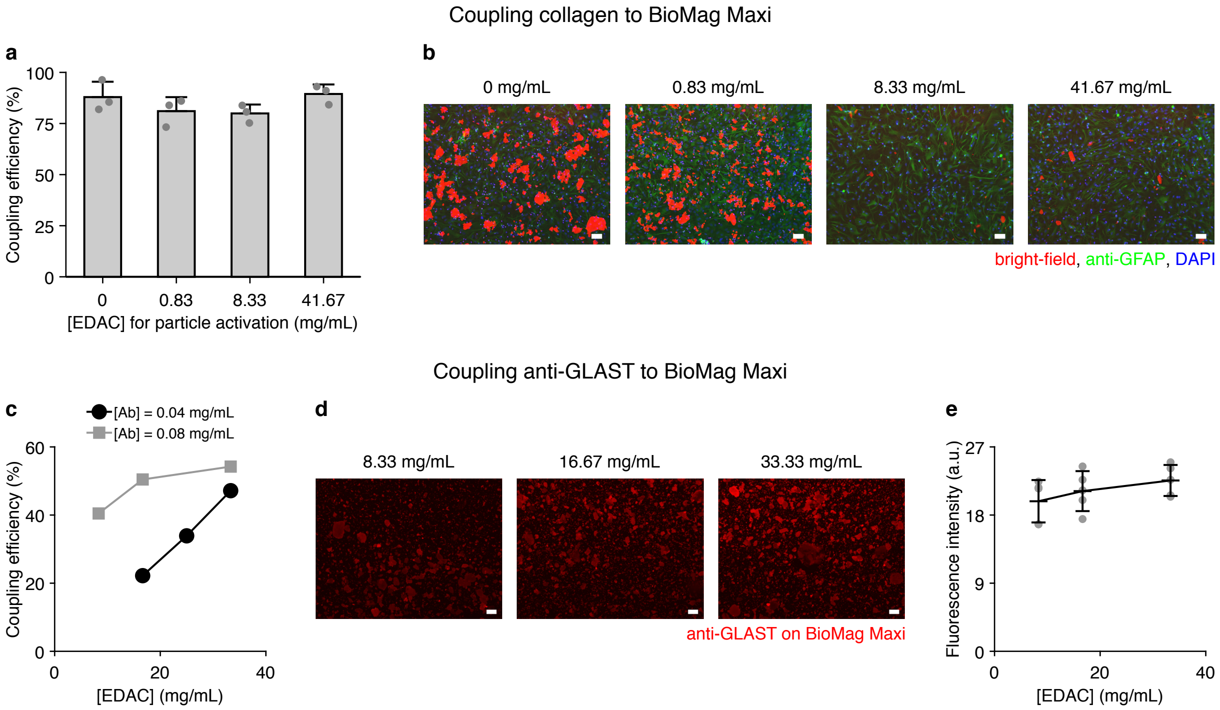

Figure S6. Optimization of the methods for coupling ligands to BioMag Maxi particles. a) The compound 1-ethyl-3-(3-dimethylaminopropyl) carbodiimide (EDAC) was used to activate BioMag Maxi particles and its concentration ([EDAC]) did not have a big influence on the coupling efficiency of collagen. n = 3 samples for each condition. b) Example micrographs showing the attachment of collagen-coupled particles to astrocytes. Exposure to high [EDAC] caused cross-linking between particles and the formation of large clumps during the preparation. When applied to astrocyte cultures, the large clumps would fail to attach firmly to the cells and be easily lost during the washing steps, resulting in the absence of particles in subsequent images. Therefore, no EDAC was used to couple collagen to BioMag Maxi particles. c) The coupling efficiency of anti-GLAST (Ab) improved as [EDAC] and the concentration of the ligand itself were increased. n = 1 sample for each condition. d) Example fluorescence images of anti-GLAST-coupled particles attached to astrocytes (astrocyte image not shown). e) Higher coupling efficiencies led to increases in fluorescence intensity of the particles. n = 5 images for each condition. Because of the results in Panel c-e, a solution with 33.33 mg mL^{-1} EDAC was used to activate BioMag Maxi particles, which were then incubated in a solution containing 0.08 mg mL^{-1} anti-GLAST. Data shown as mean ± S.D. Scale bar = 50 μm.

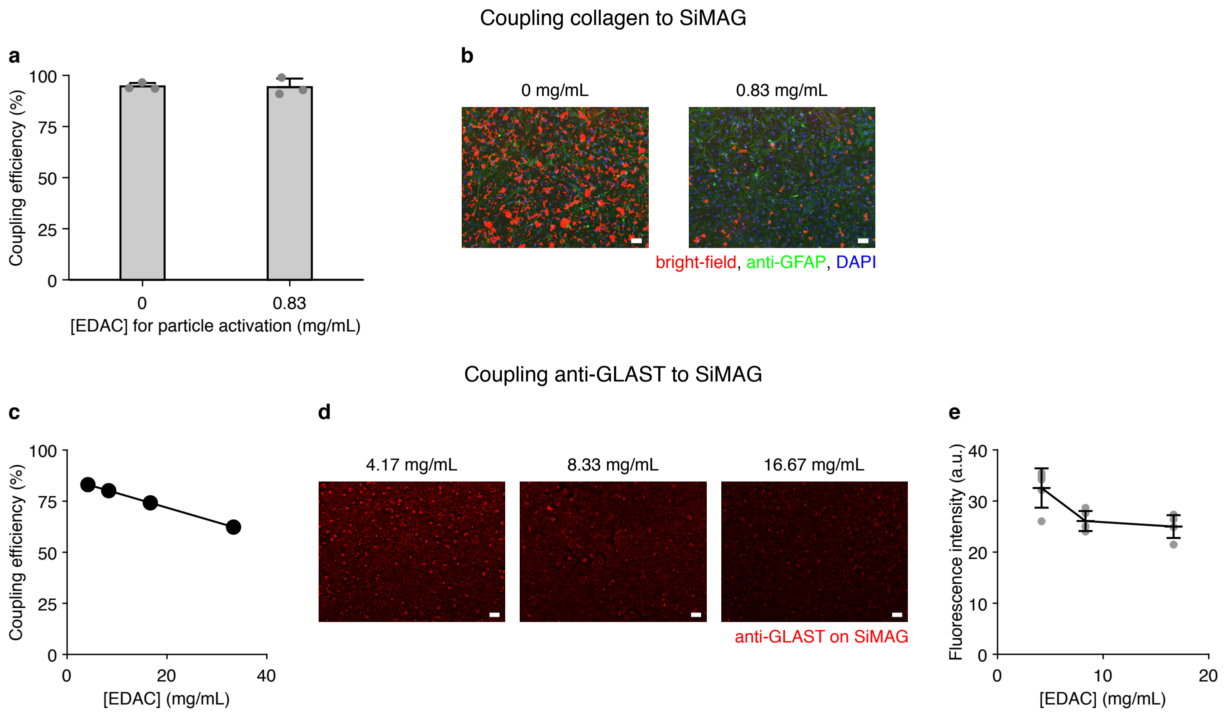

Figure S7. Optimization of the methods for coupling ligands to SiMAG particles. a) Like BioMag Maxi particles, collagen adhered to SiMAG particles with high efficiency regardless of the [EDAC] used. n = 3 samples for each condition. b) Like BioMag Maxi particles, exposure to EDAC also rendered collagen-coated SiMAG particles less able to bind to cells. Hence no EDAC was used to couple collagen to SiMAG particles. c) Unlike BioMag Maxi particles, less anti-GLAST was coupled to SiMAG particles as [EDAC] was increased. n = 1 sample for each condition. d) Example fluorescence images of anti-GLAST-coupled particles attached to astrocytes (astrocyte image not shown). e) Lower coupling efficiencies led to decreases in fluorescence intensity of the particles. n = 5 images for each condition. Because of the results in Panel c-e, a solution with 4.17 mg mL^{-1} EDAC was used to activate SiMAG particles. Data shown as mean ± S.D. Scale bar = 50 μm.

Name	iron (II,III) oxide	BioMag® Plus Carboxyl	BioMag® Maxi Carboxyl	SiMAG-Carboxyl	fluidMAG-CT
**Short Name**	Fe_3O_4	BioMag Plus	BioMag Maxi	SiMAG	fluidMAG
**Type of Iron Oxide**	Fe_3O_4	Fe_3O_4	Fe_3O_4	γ-Fe_2O_3	Fe_3O_4
**Matrix**	none	silane	silane	silica	citric acid
**Size [µm]**	< 5[a)]	~1.5[a)]	~3-12[a)]	0.5[a)]	0.1[a)]
**Density [kg m^{-3}]**	5.2E3	2.5E3[a)]	2.5E3[b)]	2.3E3[a)]	1.3E3[a)]
**Made by**	Sigma-Aldrich	Bangs Laboratories	Bangs Laboratories	chemicell GmbH	chemicell GmbH

Table S1. List of iron oxide particles used.

a) values from product data sheets. b) values assumed to be equal to those of BioMag Plus particles.

Data: $[ATP]_e$ changes (yoke magnet, 7 A, 10 mm above base, BioMag Maxi) in Figure 2b

Linear Regression Model: $[ATP]_e\_Change = \beta_0 + \beta_1(Particle\_Type) + \beta_2([Particle])$

Model Summary			
R^2	Adjusted R^2	Standard Error of the Estimate	*n*
0.143	0.124	0.426	96

ANOVA					
	Sum of Squares	df	Mean Squares	*F*	p value
Regression	2.817	2	1.408	7.754	7.683E-04
Residual	16.893	93	0.182		
Total	19.710	95	0.207		

Coefficients									
	Estimate	S.E.	*t*	one- or two-tailed	df	p value	95% CI		Cohen's *d*
Intercept	-0.473	0.144	-3.282	2	93	0.001	-0.760	-0.187	-0.335
Particle Type	0.095	0.087	1.092	2	93	0.278	-0.078	0.268	0.111
[Particle] [mg mL^{-1}]	1.489	0.394	3.784	2	93	2.734E-04	0.708	2.271	0.386

Table S2. Multiple linear regression performed on $[ATP]_e$ change measurements.

Particle type was coded as 0 = BioMag Maxi + anti-GLAST, 1 = BioMag Maxi + collagen.

Abbreviations: [Particle], the concentration of the particle suspension applied to the astrocyte culture; df, degree of freedom; S.E., standard error; CI, confidence interval.

Data: $[ATP]_e$ changes (yoke magnet, 7 A, 10 mm above base, SiMAG) in Figure 2d

Linear Regression Model: $[ATP]_e\_Change = \beta_0 + \beta_1(Particle\_Type) + \beta_2([Particle])$

Model Summary			
R^2	Adjusted R^2	Standard Error of the Estimate	*n*
0.077	0.064	0.715	144

ANOVA					
	Sum of Squares	df	Mean Squares	*F*	p value
Regression	6.003	2	3.002	5.877	0.004
Residual	72.016	141	0.511		
Total	78.019	143	0.546		

Coefficients									
	Estimate	S.E.	*t*	one- or two-tailed	df	p value	95% CI		Cohen's *d*
Intercept	-0.559	0.234	-2.384	2	141	0.018	-1.022	-0.095	-0.199
Particle Type	-0.056	0.119	-0.467	2	141	0.641	-0.291	0.180	-0.039
[Particle] [mg mL^{-1}]	1.121	0.330	3.396	2	141	8.873E-04	0.468	1.773	0.283

Table S3. Multiple linear regression performed on $[ATP]_e$ change measurements. Particle type was coded as 0 = SiMAG + anti-GLAST, 1 = SiMAG + collagen.

Data: $[ATP]_e$ changes (yoke magnet, 10 mm above base, SiMAG) in Figure 2f

Linear Regression Model:

$$[ATP]_e\_Change = \beta_0 + \beta_1(Stimulation\_Regime) + \beta_2([Particle])$$

Model Summary			
R^2	Adjusted R^2	Standard Error of the Estimate	*n*
0.006	-0.008	2.385	144

ANOVA					
	Sum of Squares	df	Mean Squares	*F*	p value
Regression	4.523	2	2.261	0.398	0.673
Residual	801.854	141	5.687		
Total	806.377	143	5.639		

Coefficients									
	Estimate	S.E.	*t*	one- or two-tailed	df	p value	95% CI		Cohen's *d*
Intercept	-0.475	0.782	-0.608	2	141	0.544	-2.022	1.071	-0.051
Stimulation Regime	0.317	0.397	0.797	2	141	0.427	-0.469	1.102	0.066
[Particle] [mg mL^{-1}]	0.441	1.101	0.401	2	141	0.689	-1.735	2.618	0.033

Table S4. Multiple linear regression performed on $[ATP]_e$ change measurements. Stimulation regime was coded as 0 = SiMAG only with yoke magnet (7 A, 10 mm above base), 1 = SiMAG + anti-GLAST with no magnetic field.

Data: $[ATP]_e$ after incubation with different SiMAG preparations for 1 h (Figure S3f)

General Linear Model: $[ATP]_e = \beta_0 + \beta_1 X_1 + \beta_2 X_2 + \beta_3 X_3 + \beta_4 X_4$

Model Summary			
R^2	Adjusted R^2	Standard Error of the Estimate	*n*
0.779	0.776	4.051	240

ANOVA					
	Sum of Squares	df	Mean Squares	*F*	p value
Regression	13651.094	3	4550.365	277.293	<1E-16
Residual	3872.755	236	16.410		
Total	17523.849	239			

Coefficients									
	Estimate	S.E.	*t*	one- or two-tailed	df	p value	95% CI		Cohen's *d*
Intercept	6.806	0.214	31.800	2	236	<1E-16	6.385	7.228	2.053
X_1 (SiMAG only)	15.850	0.501	31.640	2	236	<1E-16	14.863	16.836	2.042
X_2 (SiMAG + anti-GLAST)	-1.323	0.411	-3.220	2	236	0.001	-2.133	-0.513	-0.208
X_3 (SiMAG + collagen)	-3.796	0.447	-8.496	2	236	2.220E-15	-4.677	-2.916	-0.548
X_4 (no particle)	-3.924	0.501	-7.833	2	236	1.616E-13	-4.911	-2.937	-0.506

Two-sample *t*-tests specified by contrasts									
Test Name	Estimate	S.E.	*t*	one- or two-tailed	df	p value	95% CI		Cohen's *d*
SiMAG only > SiMAG + anti-GLAST	17.173	0.740	23.219	2	236	<1E-16	15.716	18.630	1.499
SiMAG only > SiMAG + collagen	19.646	0.773	25.399	2	236	<1E-16	18.122	21.170	1.640
SiMAG only > no particle	19.773	0.827	23.913	2	236	<1E-16	18.144	21.402	1.544
SiMAG + anti-GLAST > SiMAG + collagen	2.473	0.679	3.641	2	236	3.342E-04	1.135	3.812	0.235
SiMAG + anti-GLAST > no particle	2.601	0.740	3.516	2	236	0.001	1.144	4.058	0.227
SiMAG + collagen > no particle	0.127	0.773	0.165	2	236	0.869	-1.396	1.651	0.011

Table S5. General linear model to predict [ATP] in the medium.

X_1: 1 = SiMAG only, 0 = all others. X_2: 1 = SiMAG + anti-GLAST, 0 = all others. X_3: 1 = SiMAG + collagen, 0 = all others. X_4: 1 = No particle, 0 = all others.

Data: $[ATP]_e$ changes (yoke magnet or Magnetic Mangle, Fe_3O_4 + collagen) in Figure S4d

Linear Regression Model: $[ATP]_e\_Change = \beta_0 + \beta_1(Median\_Stress)$

Model Summary						
Pearson's *r*	95% CI for *r*		R^2	Adjusted R^2	Standard Error of the Estimate	*n*
0.700	0.413	0.860	0.490	0.467	6.390	24

ANOVA	Sum of Squares	df	Mean Squares	*F*	p value
Regression	863.237	1	863.237	21.140	1.401E-04
Residual	898.343	22	40.834		
Total	1761.579	23	76.590		

Coefficients	Estimate	S.E.	*t*	one- or two-tailed	df	p value	95% CI		Cohen's *d*
Intercept	-4.139	2.594	-1.596	2	22	0.125	-9.518	1.241	-0.326
Median Stress [Pa]	11.275	2.452	4.598	2	22	1.401E-04	6.189	16.361	0.939

Table S6. Linear regression performed on $[ATP]_e$ change measurements.

Data: $[ATP]_e$ changes (yoke magnet or Magnetic Mangle, SiMAG + anti-GLAST, Figure 3g)

Linear Regression Model: $[ATP]_e\_Change = \beta_0 + \beta_1(Median\_Stress)$

Model Summary						
Pearson's r	95% CI for r		R^2	Adjusted R^2	Standard Error of the Estimate	n
0.310	0.138	0.463	0.096	0.088	1.602	120

ANOVA					
	Sum of Squares	df	Mean Squares	F	p value
Regression	32.107	1	32.107	12.505	5.809E-04
Residual	302.966	118	2.568		
Total	335.073	119	2.816		

Coefficients									
	Estimate	S.E.	t	one- or two-tailed	df	p value	95% CI		Cohen's d
Intercept	0.084	0.262	0.321	2	118	0.749	-0.435	0.603	0.029
Median Stress [Pa]	2.312	0.654	3.536	2	118	5.809E-04	1.017	3.607	0.323

Table S7. Linear regression performed on $[ATP]_e$ change measurements.

Data: $[ATP]_e$ changes (yoke magnet or Magnetic Mangle, SiMAG) in Figure 3i

Linear Regression Model:

$$[ATP]_e\_Change = \beta_0 + \beta_1(Particle\_Type) + \beta_2(Median\_Stress)$$

Model Summary			
R^2	Adjusted R^2	Standard Error of the Estimate	*n*
0.042	0.032	3.618	192

ANOVA					
	Sum of Squares	df	Mean Squares	*F*	p value
Regression	108.341	2	54.171	4.138	0.017
Residual	2474.109	189	13.091		
Total	2582.451	191	13.521		

Coefficients									
	Estimate	S.E.	*t*	one- or two-tailed	df	p value	95% CI		Cohen's *d*
Intercept	0.747	0.575	1.299	2	189	0.196	-0.387	1.882	0.094
Particle Type	-1.474	0.522	-2.823	2	189	0.005	-2.504	-0.444	-0.204
Median Stress [Pa]	0.644	1.158	0.556	2	189	0.579	-1.640	2.927	0.040

Table S8. Multiple linear regression performed on $[ATP]_e$ change measurements. Particle type was coded as 0 = SiMAG + anti-GLAST, 1 = unmodified SiMAG.

	Caudal distance from Bregma [mm]	Lateral distance from Bregma [mm]	Depth from brain surface [mm]
Large rats (270-310 g)	11.8	1.7	8.5
	11.8	-1.7	8.5
Small rats (80-120 g)	10.7	1.5	8.0
	10.7	-1.5	8.0

Table S9. Stereotaxic coordinates for intracranial injections into the VLM of rats.
